# supraHex: An R/Bioconductor package for tabular omics data analysis using a supra-hexagonal map^☆^

**DOI:** 10.1016/j.bbrc.2013.11.103

**Published:** 2014-01-03

**Authors:** Hai Fang, Julian Gough

**Affiliations:** Computational Genomics Group, Department of Computer Science, University of Bristol, Merchant Venturers Building, Bristol BS8 1UB, UK

**Keywords:** Bioinformatics, Clustering, Sample correlation, Visualisation, DNA replication timing, Gene expression

## Abstract

Biologists are increasingly confronted with the challenge of quickly understanding genome-wide biological data, which usually involve a large number of genomic coordinates (e.g. genes) but a much smaller number of samples. To meet the need for data of this shape, we present an open-source package called ‘supraHex’ for training, analysing and visualising omics data. This package devises a supra-hexagonal map to self-organise the input data, offers scalable functionalities for post-analysing the map, and more importantly, allows for overlaying additional data for multilayer omics data comparisons. Via applying to DNA replication timing data of mouse embryogenesis, we demonstrate that supraHex is capable of simultaneously carrying out gene clustering and sample correlation, providing intuitive visualisation at each step of the analysis. By overlaying CpG and expression data onto the trained replication-timing map, we also show that supraHex is able to intuitively capture an inherent relationship between late replication, low CpG density promoters and low expression levels. As part of the Bioconductor project, supraHex makes accessible to a wide community in a simple way, what would otherwise be a complex framework for the ultrafast understanding of any tabular omics data, both scientifically and artistically. This package can run on Windows, Mac and Linux, and is freely available together with many tutorials on featuring real examples at http://supfam.org/supraHex.

## Highlights

• supraHex is an open-source R/Bioconductor package for tabular omics data analysis.
• A supra-hexagonal map is designed to self-organise omics data.
• The supraHex map analyses both genes and samples at the same time.
• The supraHex map can be overlaid by additional data for multilayer omics data comparisons.
• supraHex can tell inherent relations between replication timing, CpG and expression.

## 1 Introduction

Conventional microarray platforms [1], [2] and recent next-generation sequencing technologies [3], [4] are producing ever-increasing amounts of omics data. The forms of raw data may differ in the preprocessing as required, but the downstream (analysable) data can universally be stored in a table-like matrix, which digitises expression levels or other bioactivities of genomic coordinates (e.g. genes for simplicity) across the samples. This tabular matrix of genes × samples usually contains a large number of genes (i.e. large *p* features) but a much smaller number of samples (i.e. small *n* samples). This ‘large *p*, small *n*’ data analysis challenge [5] is a common barrier to biologists in checking and exploring their own omics data. Sanity checking and an initial quick exploration are essential first steps on the route to an eventual downstream discovery and final interpretation. A long list of genes requires that dimension of the information to be compressed, e.g. via clustering. The clustering of genes still needs to be visualised, done by projection onto a 2-dimensional (2D) space. A self-organising learning algorithm [6] is well-suited for this purpose as it imposes an orderly structure on the clusters.

**Fig. 1:**
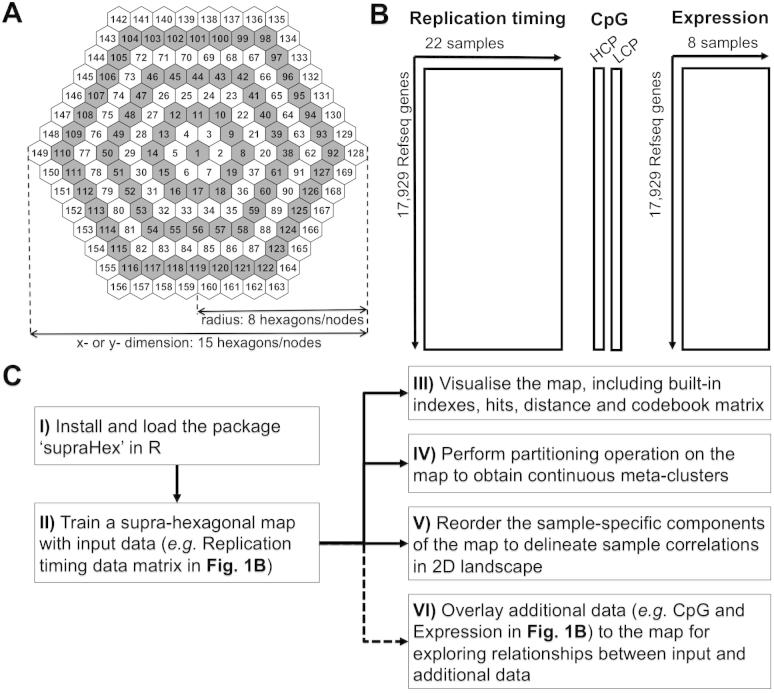
The key functionalities in supraHex. (A) Architectural design of a supra-hexagonal map with node numbering. It has a total of 169 smaller hexagons (i.e. map nodes) that are indexed as follows: start from the center, and then expand circularly outwards, and for each circle increase in an anti-clockwise order. The radius or the *x*- or *y*-dimension of the map grid uniquely determines this architecture. (B) Illustration of datasets used to demonstrate the functionalities of supraHex. DNA replication timing data matrix during mouse embryogenesis is used as input data for training the supra-hexagonal map. CpG data and expression data matrices are used for overlaying onto the trained replication-timing map. CpG data contains information on gene promoter CpG classification: HCP stands for the vector with 1 for a high CpG density promoter and 0 otherwise; LCP for the vector with 1 for a low CpG density promoter and 0 otherwise. Unlike the logical values in CpG data, expression data contains numeric expression levels for genes. (C) Schematic flowchart of the supraHex workflow. After being installed and loaded, the package takes as input any tabular data matrix and outputs the trained supra-hexagonal map and its associated codebook matrix. The trained map can be post-processed for multiple purposes, including: (III) visualisation of various properties associated with the map, (IV) map partitioning for obtaining gene meta-clusters, (V) sample-specific component reordering for sample correlation, and (VI) if provided, additional data can also be overlaid onto the trained map for exploring relationships between input and additional data.

Commonly the structure imposed for visualising a 2-D map is a square grid, but a giant hexagon formed by smaller hexagons is prevalent in many natural and man-made objects, such as a honeycomb or at Giant’s Causeway. Inspired by this, we devised a supra-hexagonal map that seamlessly consists of smaller hexagons. It has symmetric beauty around the center, from which individual hexagons radiate outwards (Fig. 1A); this makes the supra-hexagonal map suitable for modelling symmetric data, particularly omics data. Omics data reveal biological information on a global scale, and the rationale behind data normalisation [7] is that most genes do not change or do so randomly. In other words, most genes will map to the centre with radial symmetry, giving a visual normalisation against which non-random changes stand out. To make use of this symmetry of the gene-sample matrix in high-dimensional input space, we use a self-organising learning algorithm but based on the supra-hexagonal layout. This is one of the key functionalities of the package ‘supraHex’. This package produces a map in which: (i) genes with similar data patterns self-organise to the same or nearby nodes in the map, and (ii) the distribution of genes across the 2D map is representative of the high-dimensional input space. Also, supraHex can be applied to multilayer omics datasets (such as in Fig. 1B). In this paper we demonstrate that supraHex makes it easy to carry out integrated tasks such as: gene clustering/meta-clustering, sample correlations and visualisations, and the overlaying of additional data onto the map (Fig. 1C). As an open-source R package, supraHex is distributed as part of the Bioconductor project [8].

## 2 Materials and methods

### 2.1 The supra-hexagonal map trained via a self-organising learning algorithm

The package ‘supraHex’ is a pure R implementation of a self-organising learning algorithm [6] applied to the symmetric topology of the supra-hexagonal map. For details on the algorithm as well as the topology and training tailored to this architectural design, the reader is referred to the Reference Manual (Supplementary material 1). The package takes as input a matrix of values for genes versus samples, and sets up the pipeline for the learning process: initialisation, training and many auxiliary functions. The output of the learning is the mapping of similar input data onto neighboring regions of the supra-hexagonal map. Each map node is associated with two coordinates: one in 2D output space (i.e. what we can see), and the other in high-dimensional input space (i.e. what we can imagine; represented as prototype vectors with the same dimension as the input data vectors). Prototype vectors in the map nodes collectively constitute a so-called codebook matrix. So in essence, supraHex converts the gene-sample matrix into the codebook matrix that is associated with the supra-hexagonal map.

### 2.2 Visualisations at and across nodes of the map

supraHex provides several options for visualising the map generated from the training process. These visualisations fall into two general categories: across nodes and within nodes. The first is to visualise the single-value properties associated with each map node; each node (hexagon) is numbered (as shown and explained in Fig. 1A) so that it can easily be referred to. The map distance tells how far each map node is away from its neighbors in high-dimensional input space, while the ‘hit’ histogram over the map shows how many input data vectors are best hitting each node. The second type of visualisation is for illustrating the patterns (in the prototype vectors) associated with each map node, such as those stored in the codebook matrix. The patterns can be visualised by line plots and bar plots. When a pattern spans both negative and positive values, the zero axis is also displayed. The Reference Manual contains advanced-usage instructions for customising the plotting of patterns.

### 2.3 Partitioning of the map into gene meta-clusters

As well as clustering genes into each node (hexagon), supraHex is also able to perform a partitioning operation on the trained map to obtain gene meta-clusters covering regions of the map. The idea is to first identify local minima of distances between map nodes, and treat these as seeds to group the remaining nodes into meta-clusters that cover the whole map. Starting from the seeds, each remaining node can either simply be grouped with the best-matching seed, or the partitioning can be done using the region-growing algorithm proposed in [9]. The purpose of the region-growing algorithm is to ensure that each meta-cluster is continuous over the map. For visualising meta-clusters, the map is colour-coded by region with the seeds highlighted as well.

### 2.4 Sample correlation

**Fig. 2:**
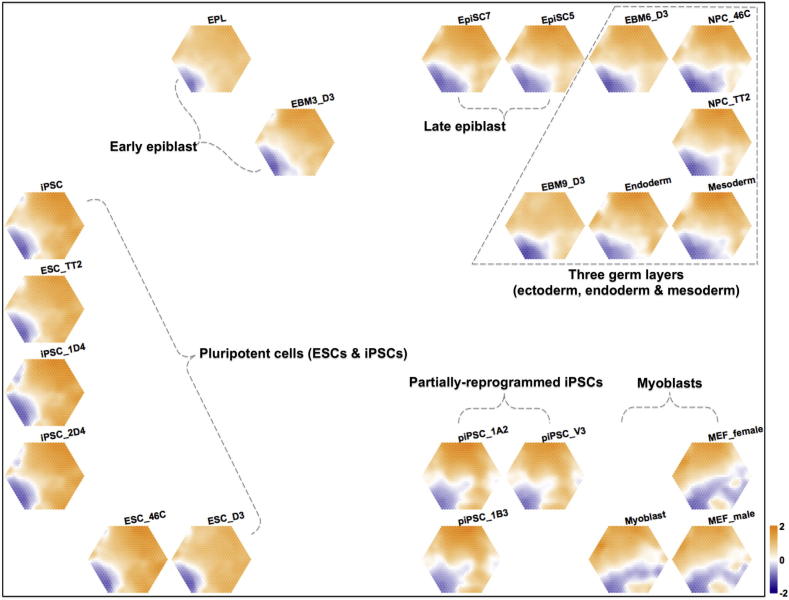
The replication timing map of 22 cell types from mouse embryogenesis. After the supra-hexagonal map is trained on replication timing data, the codebook matrix associated with the trained map is used for sample-specific component reordering and visualisation. Each map illustrates a sample-specific replication-timing profile. The colour bar represents replication-timing ratios [=log2(Early/Late)], with orange for early replication and blue for late replication. Across components/samples, genes with similar replication-timing patterns are mapped onto the same position in the map. The outermost frame represents the replication-timing landscape for the samples analysed, from which geometric locations of components/samples delineate relationships between these 22 cell types. They are: (i) pluripotent cells, including ESCs (embryonic stem cells) and iPSCs (induced pluripotent stem cells), (ii) early epiblast, (iii) late epiblast, (iv) three germ layers, including ectoderm, mesoderm and endoderm, (v) partially-reprogrammed iPSCs (piPSCs) and (vi) differentiated myoblasts. (For interpretation of the references to colour in this figure legend, the reader is referred to the web version of this article.)

**Fig. 3:**
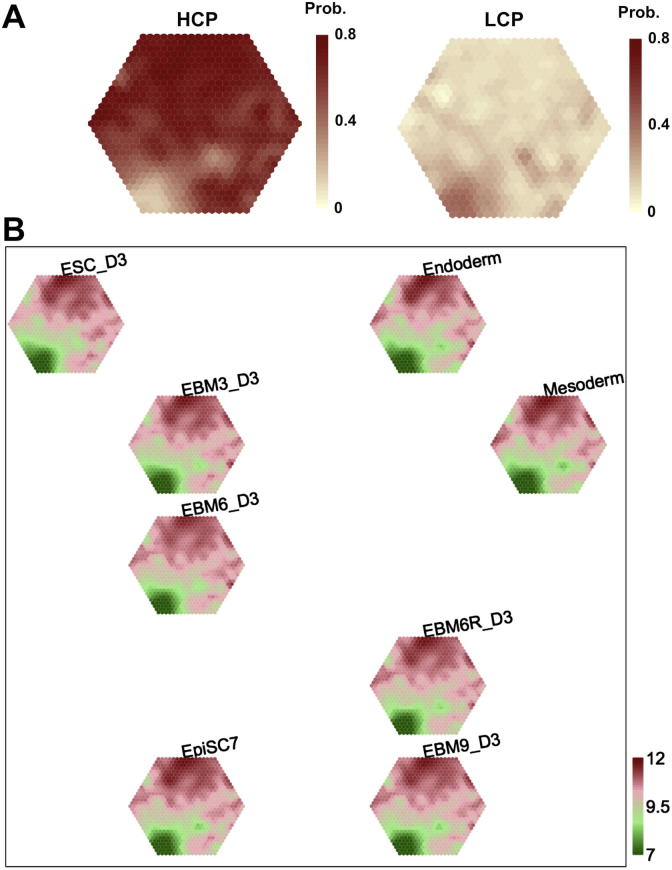
Using the replication-timing map to intuitively explore relationships between replication timing, CpG and expression. (A) CpG data overlaid onto the replication-timing map. Left: the HCP vector with 1 for high CpG density promoters and 0 otherwise; Right: the LCP vector with 1 for low CpG density promoters and 0 otherwise. The colour bar indicates the probability of containing genes with HCPs (left panel) or LCPs (right panel); the stronger, the higher. (A) Expression data overlaid onto the replication-timing map. After the replication-timing map is overlaid with expression data, the codebook matrix associated with the overlaid expression map is used for sample-specific component reordering and visualisation. Each map illustrates a sample-specific expression profile. The colour bar stands for expression levels [=log2(Intensity)], with dark red for high expression and dark green for low expression. It should be noted that here genes with similar replication-timing patterns are still mapped onto the same position on the map, but the illustrations are replaced with expression patterns. (For interpretation of the references to colour in this figure legend, the reader is referred to the web version of this article.)

The concept of using supraHex for visualising sample correlation is to lay out the samples on a new 2D square lattice (as in the examples shown in Figs. 2 and 3B). This can be done based on either the sample-wise vectors of the codebook/input matrix, or on the covariance matrix thereof. supraHex provides a choice for calculation of the covariance matrix based on a variety of different distance metrics. These include: some commonly used metrics (e.g. Pearson correlation, Euclidean and cityblock distance, and cosine similarity); some rank-based correlations (i.e. Spearman rho and Kendall tau); and mutual information as a general measure of dependency. Using the covariance matrix can vastly reduce the cost of training the layout, which is essential when the input data matrix is prohibitively large. Samples are organised such that those with similar profiles are closer to each other within this layout (called the ‘sample landscape’). Within this landscape, the geometric locations of samples delineate their relationships.

### 2.5 Overlaying additional data onto the trained map for exploring relationships between input and additional data

The overlaying of additional data enables comparisons of multilayer omics data. In the example shown in Fig. 1B, the input data used for training are DNA replication timing, and the two additional sources to be overlaid are CpG data and expression data. The overlaying onto each map node is realised by: first calculating the neighborhood-kernel-weighted hits/counts of the input data, then calculating the accumulated values (similarly weighted by the neighborhood kernel) of the additional data to be mapped onto that node, and finally obtaining the overlaid values via normalisation of the accumulated values by hits. The reason for weighting by the neighborhood kernel is to avoid rigid overlaying which could result from focusing on the best-matching map nodes only; there may exist several closest best-matching nodes for an input data vector.

### 2.6 Public accessibility of the package and tutorial-like applications on real cases

The package ‘supraHex’ is regularly maintained and freely available via the Bioconductor website (http://bioconductor.org). Along with the package, there are a detailed description of the package functions in the Reference Manual (Supplementary material 1) and a task-oriented description of the package functionalities in the User Manual (see Supplementary material 2). In addition to this, a dedicated webpage (http://supfam.org/supraHex) provides applications to many real cases including the example presented in the following Results section. On the website the reader can find the workflow of R commands used in each case (and the corresponding results/visuals). The users are encouraged to reproduce and adapt these workflows for analysing their own omics datasets.

## 3 Results

To showcase the multifaceted functionalities supported by supraHex (Fig. 1), we used published multilayer omics datasets on DNA replication timing, promoter CpG classification and gene expression [10], [11]. These datasets consist of digitised replication timing, promoter CpG status and expression levels of 17,292 genes in a variety of samples. We first chose the DNA replication timing data matrix as the input for training a supra-hexagonal map. As a result of step (V) in Fig. 1C, the reordering and visualisation of sample-specific components of the map are able to reveal intuitive information about both genes and samples (see Fig. 2). Comparing the supraHex output to the heatmap display shown in the original publication [11], it can be seen that several features associated with input replication-timing can be revealed more intuitively in the output from the supraHex analysis. Firstly, genes with early replication timing outnumber those with late replication timing, irrespective of the cell type analysed. Secondly, early-replicated genes are mostly mapped onto the top part of the map, while the late-replicated genes are consistently clustered in the bottom-left corner of the map. Thirdly, cell types of the same category share similar replication-timing profiles/signatures, and are thus placed together at a specific location of this 2D landscape (framed by the outermost black lines). Simultaneous inspection of the genes within the supra-hexagonal map and the samples within the landscape reveals: (i) what constitutes the replication-timing signature common to a category of cell types (such as pluripotent cells), and (ii) how signatures change across these different cell type categories.

Next, we overlaid CpG data onto the trained replication-timing map. CpG data are vectors containing logical information (Fig. 1B). The HCP vector indicates whether or not a gene contains a high CpG density promoter (HCP), while the LCP vector suggests the presence of a low CpG density promoter (LCP). The left panel in Fig. 3A is the result after overlaying the HCP vector onto the replication-timing map, showing the probability that nodes (and genes thereof) will have an HCP. In Fig. 2 it is evident that consistently late replications (in the bottom-left corner) are mostly devoid of HCP. In contrast, when overlaying the LCP vector, we see that consistently late replications are highly indicative of the presence of LCPs (the right panel in Fig. 3A).

A more general kind of data for overlaying are the expression data in Fig. 1B. In this context, the replication-timing map overlaid with the expression data produces an expression map. This expression map is also artificially associated with codebook matrix but it has the dimension of the overlaid (expression) data rather than the input (replication timing) data. As before, this artificial codebook matrix can be used for sample-specific component reordering and visualisation (see Fig. 3B). Remarkably, purely based on the overlaid expression map, two features become clear. Firstly, we can see that consistently late replications (in the bottom-left corner) consistently have low expression across samples. Secondly, the reordering of sample-specific components recovers some known relationships, such as the sequence of neural differentiation of the embryonic stem cell line D3 (the path from ESC_D3 via EBM3_D3 and EBM6_D3 to EBM9_D3). Taking into account the fact that the additional data were not used in the training of the map, the information revealed in Fig. 3 is truly reflective of the inherent relationships between late replication, low CpG density promoters and low expression levels.

## 4 Discussion

The open-source package ‘supraHex’, our contribution to the Bioconductor project, is intended to meet the challenging demand for a way to intuitively and quickly understanding omics data. The concept of self-organising omics data is not new [12] but nonetheless fascinating that even today it remains a powerful technique [13], [14]. However, previous tools (such as somtoolbox [15], Kohonen [16] and Cluster3.0 [17]) compromise on availability, visual novelty, or functional scalability (see Table 1 for a comparison). Instead, supraHex is tailored to the new challenges of omics data analysis. It is easy to use, visually friendly at all steps of the analysis, and most importantly, provides self-explanatory and reproducible results. As demonstrated here, the map produced by supraHex can be used to analyse both genes and samples at the same time, and can also be overlaid with additional data for multilayer omics data comparisons. Being an R/Bioconductor package, we anticipate that supraHex will deliver to a very broad bioinformatics community an intuitive and ultrafast understanding of any tabular omics data, both scientifically and aesthetically.

**Table 1:** Comparison to other tools implementing self-organising learning algorithms.

Features	supraHex	somtoolbox	Kohonen	Cluster3.0
Programming language	R	Matlab^a^	R	C
Map shape	supra-hexagon	Sheet^b^	Sheet	Sheet
Visually friendly	Yes	Yes	Yes	No
With neighbour kernels	Yes	Yes	No	No
Meta-clustering	Yes	Yes	No	No
Sample reordering	Yes	Yes	No	No
Overlaying with additional data	Yes	No	No	No
Bioconductor project	Yes	No	No	No

a Needs commercial license.

b Also supports the cylinder and toroid shapes but are less popular.

## Appendix A Supplementary data

Supplementary data 1 This document contains supplementary materials.

Supplementary data 2 This document contains supplementary materials.
