# Supplementary material for: supraHex: An R/Bioconductor package for tabular omics data analysis using a supra-hexagonal map
Source: Biochem Biophys Res Commun. 2014 Jan 3;443(1):285–9. doi: 10.1016/j.bbrc.2013.11.103 (PMC3905187; doi:10.1016/j.bbrc.2013.11.103)
Supplement: Supplementary data 1 — This document contains supplementary materials. [file mmc1.pdf]

Package ‘supraHex’

November 4, 2013

Type Package

Title A supra-hexagonal map for analysing tabular omics data

Version 1.1.3

Date 2013-11-4

Author Hai Fang and Julian Gough

Maintainer Hai Fang <hfang@cs.bris.ac.uk>

Depends R (>= 3.0.1), hexbin

Imports grid, MASS, Biobase

Description A supra-hexagonal map is a giant hexagon on a 2-dimensional grid seamlessly consisting of smaller hexagons. It is supposed to train, analyse and visualise a high-dimensional omics input data. The supraHex is able to carray out gene clustering/meta-clustering and sample correlation, plus intuitive visualisations to facilitate exploratory analysis. More importantly, it allows for overlaying additional data to explore relations between input and additional data. Uniquely to this package, users can ultrafastly understand any tabular omics data, both scientifically and artistically, especially in a sample-specific fashion but without loss of information on large genes.

URL http://supfam.org/SUPERFAMILY/dcGO/supraHex.html

Collate sHexGrid.r sTopology.r sInitial.r sTrainology.r sTrainSeq.r sTrainBatch.r sBMH.r sPipeline.r sNeighDirect.r sNeighAny.r sHexDist.r sDistance.r sDmat.r sDmatMinima.r sDmatCluster.r sCompReorder.r sWriteData.r sMapOverlay.r visHexGrid.r visHexMapping.r visHexComp.r visColormap.r visColorbar.r visVp.r visHexMulComp.r visCompReorder.r visHexPattern.r visDmatCluster.r visKernels.r

License GPL-2

biocViews Bioinformatics, Clustering, Visualization, GeneExpression

R topics documented:

Fang 2
Golub 3
sBMH 3

sCompReorder 5
sDistance 7
sDmat 8
sDmatCluster 9
sDmatMinima 10
sHexDist 11
sHexGrid 12
sInitial 14
sMapOverlay 15
sNeighAny 16
sNeighDirect 17
sPipeline 18
sTopology 20
sTrainBatch 22
sTrainology 24
sTrainSeq 26
sWriteData 27
visColorbar 29
visColormap 30
visCompReorder 31
visDmatCluster 32
visHexComp 33
visHexGrid 35
visHexMapping 36
visHexMulComp 37
visHexPattern 38
visKernels 40
visVp 41
Xiang 42

Index **43**

`Fang` *Human embryo gene expression dataset from Fang et al. (2010)*

Description

Human embryo dataset contains gene expression levels (5441 genes and 18 embryo samples) from Fang et al. (2010).

Usage

```
data(Fang)
```

Value

`Fang` a gene expression matrix of 5441 genes x 18 samples, involving six successive stages, each with three replicates.

`Fang.sampleinfo` a matrix containing the information of the 18 samples for the expression matrix `Fang`. The three columns correspond to the sample `Name`, `Stage`, and `Replicate`, respectively.

`Fang.geneinfo` a matrix containing the information of the 5441 genes for the expression matrix `Fang`. The four columns correspond to the gene `AffyID`, `EntrezGene`, and `Symbol`, respectively.

References

Fang et al. (2010). Transcriptome analysis of early organogenesis in human embryos. *Developmental Cell*, 19(1):174-84.

`Golub` *Leukemia gene expression dataset from Golub et al. (1999)*

Description

Leukemia dataset (learning set) contains gene expression levels (3051 genes and 38 patient samples) from Golub et al. (1999). This dataset has been pre-processed: capping into floor of 100 and ceiling of 16000; filtering by exclusion of genes with $max/min <= 5$ or $max - min <= 500$, where max and min refer respectively to the maximum and minimum intensities for a particular gene across mRNA samples; 2-base logarithmic transformation.

Usage

```
data(Golub)
```

Value

`Golub` a gene expression matrix of 3051 genes x 38 samples. These samples include 11 acute myeloid leukemia (AML) and 27 acute lymphoblastic leukemia (ALL) which can be further subtyped into 19 B-cell ALL and 8 T-cell ALL.

References

Golub et al. (1999). Molecular classification of cancer: class discovery and class prediction by gene expression monitoring, *Science*, Vol. 286:531-537. http://www-genome.wi.mit.edu/MPR/

`sBMH` *Function to identify the best-matching hexagons/rectangles for the input data*

Description

`sBMH` is supposed to identify the best-matching hexagons/rectangles (BMH) for the input data.

Usage

```
sBMH(sMap, data, which_bmh = c("best", "worst", "all"))
```

Arguments

sMap	an object of class "sMap" or a codebook matrix
data	a data frame or matrix of input data
which_bmh	which BMH is requested. It can be a vector consisting of any integer values from [1, nHex]. Alternatively, it can also be one of "best", "worst" and "all" choices. Here, "best" is equivalent to 1, "worst" for $nHex$, and "all" for $seq(1, nHex)$

Value

a list with following components:

bmh	the requested BMH matrix of dlen x length(which_bmh), where dlen is the total number of rows of the input data
qerr	the corresponding matrix of quantization errors (i.e., the distance between the input data and their BMH), with the same dimensions as "bmh" above
mqe	the mean quantization error for the "best" BMH
call	the call that produced this result

Note

"which_bmh" upon request can be a vector consisting of any integer values from [1, nHex]

See Also

sPipeline

Examples

```
# 1) generate an iid normal random matrix of 100x10
data <- matrix( rnorm(100*10,mean=0,sd=1), nrow=100, ncol=10)

# 2) from this input matrix, determine nHex=5*sqrt(nrow(data))=50,
# but it returns nHex=61, via "sHexGrid(nHex=50)", to make sure a supra-hexagonal grid
sTopol <- sTopology(data=data, lattice="hexa", shape="suprahex")

# 3) initialise the codebook matrix using "uniform" method
sI <- sInitial(data=data, sTopol=sTopol, init="uniform")

# 4) define trainology at "rough" stage
sT_rough <- sTrainology(sMap=sI, data=data, stage="rough")

# 5) training at "rough" stage
sM_rough <- sTrainSeq(sMap=sI, data=data, sTrain=sT_rough)

# 6) define trainology at "finetune" stage
sT_finetune <- sTrainology(sMap=sI, data=data, stage="finetune")

# 7) training at "finetune" stage
sM_finetune <- sTrainSeq(sMap=sM_rough, data=data, sTrain=sT_rough)

# 8) find the best-matching hexagons/rectangles for the input data
response <- sBMH(sMap=sM_finetune, data=data, which_bmh="best")
```

sCompReorder *Function to reorder component planes*

Description

sCompReorder is supposed to reorder component planes for the input map/data. It returns an object of class "sReorder". It is realized by using a new map grid (with sheet shape consisting of a rectangular lattice) to train component plane vectors (either column-wise vectors of codebook/data matrix or the covariance matrix thereof). As a result, similar component planes are placed closer to each other. It is highly recommend to use trained map (i.e. codebook matrix) as input if data matrix is hugely big to save computational costs.

Usage

```
sCompReorder(sMap, xdim = NULL, ydim = NULL,
  amplifier = NULL,
  metric = c("none", "pearson", "spearman", "kendall", "euclidean", "manhattan", "cos", "mi"),
  init = c("linear", "uniform", "sample"),
  algorithm = c("sequential", "batch"),
  alphaType = c("invert", "linear", "power"),
  neighKernel = c("gaussian", "bubble", "cutgaussian", "ep", "gamma"))
```

Arguments

sMap	an object of class "sMap" or input data frame/matrix
xdim	an integer specifying x-dimension of the grid
ydim	an integer specifying y-dimension of the grid
amplifier	an integer specifying the amplifier (3 by default) of the number of component planes. The product of the component number and the amplifier constitutes the number of rectangles in the sheet grid
metric	distance metric used to difine the similarity between component planes. It can be "none", which means directly using column-wise vectors of codebook/data matrix. Otherwise, first calculate the covariance matrix from the codebook/data matrix. The distance metric used for calculating the covariance matrix between component planes can be: "pearson" for pearson correlation, "spearman" for spearman rank correlation, "kendall" for kendall tau rank correlation, "euclidean" for euclidean distance, "manhattan" for cityblock distance, "cos" for cosine similarity, "mi" for mutual information. See sDistance for details
init	an initialisation method. It can be one of "uniform", "sample" and "linear" initialisation methods
algorithm	the training algorithm. Currently, only "sequential" algorithm has been implemented
alphaType	the alpha type. It can be one of "invert", "linear" and "power" alpha types
neighKernel	the training neighbor kernel. It can be one of "gaussian", "bubble", "cutgaussian", "ep" and "gamma" kernels

Value

an object of class "sReorder", a list with following components:

- `nHex` — the total number of rectanges in the grid
- `xdim` — x-dimension of the grid
- `ydim` — y-dimension of the grid
- `uOrder` — the unique order/placement for each component plane that is reordered to the "sheet"-shape grid with rectangular lattice
- `coord` — a matrix of nHex x 2, with each row corresponding to the coordinates of each "uOrder" rectangle in the 2D map grid
- `call` — the call that produced this result

Note

To ensure the unique placement, each component plane mapped to the "sheet"-shape grid with rectangular lattice is determinied iteratively in an order from the best matched to the next compromised one. If multiple compoments are hit in the same rectangular lattice, the worse one is always sacrificed by moving to the next best one till all components are placed somewhere exclusively on their own.

See Also

`sTopology`, `sPipeline`, `sBMH`, `sDistance`, `visCompReorder`

Examples

```
# 1) generate an iid normal random matrix of 100x10
data <- matrix( rnorm(100*10,mean=0,sd=1), nrow=100, ncol=10)
colnames(data) <- paste(rep(S,10), seq(1:10), sep="")

# 2) get trained using by default setup
sMap <- sPipeline(data=data)

# 3) reorder component planes in different ways
# 3a) directly using column-wise vectors of codebook matrix
sReorder <- sCompReorder(sMap=sMap, amplifier=2, metric="none")
# 3b) according to covariance matrix of pearson correlation of codebook matrix
sReorder <- sCompReorder(sMap=sMap, amplifier=2, metric="pearson")
# 3c) directly using column-wise vectors of input matrix
sReorder <- sCompReorder(sMap=data, amplifier=2, metric="none")
# 3d) according to covariance matrix of pearson correlation of input matrix
sReorder <- sCompReorder(sMap=data, amplifier=2, metric="pearson")

# 4) visualise multiple component planes reorded within a sheet-shape rectangle grid
visCompReorder(sMap=sMap, sReorder=sReorder, margin=rep(0.1,4), height=7,
title.rotate=0, title.xy=c(0.45, 1), colormap="gbr", ncolors=10, zlim=c(-1,1),
border.color="transparent")
```

sDistance — *Function to compute the pairwise distance for a given data matrix*

Description

`sDistance` is supposed to compute and return the distance matrix between the rows of a data matrix using a specified distance metric

Usage

```
sDistance(data,
  metric = c("pearson", "spearman", "kendall", "euclidean", "manhattan", "cos", "mi"))
```

Arguments

`data`	a data frame or matrix of input data
`metric`	distance metric used to distance metric. See 'Note' below for options available

Value

`dist`	a symmetric distance matrix of nRow x nRow, where nRow is the number of rows of input data matrix

Note

The distance metrics are supported:

- "pearson": Pearson correlation. Note that two curves that have identical shape, but different magnitude will still have a correlation of 1
- "spearman": Spearman rank correlation. As a nonparametric version of the pearson correlation, it calculates the correlation between the ranks of the data values in the two vectors (more robust against outliers)
- "kendall": Kendall tau rank correlation. Compared to spearman rank correlation, it goes a step further by using only the relative ordering to calculate the correlation. For all pairs of data points (x_i, y_i) and (x_j, y_j), it calls a pair of points either as concordant (Nc in total) if $(x_i - x_j) * (y_i - y_j) > 0$, or as discordant ($Nd$ in total) if $(x_i - x_j) * (y_i - y_j) < 0$. Finally, it calculates gamma coefficient $(Nc - Nd)/(Nc + Nd)$ as a measure of association which is highly resistant to tied data
- "euclidean": Euclidean distance. Unlike the correlation-based distance measures, it takes the magnitude into account (input data should be suitably normalized
- "manhattan": Cityblock distance. The distance between two vectors is the sum of absolute value of their differences along any coordinate dimension
- "cos": Cosine similarity. As an uncentered version of pearson correlation, it is a measure of similarity between two vectors of an inner product space, i.e., measuring the cosine of the angle between them (using a dot product and magnitude)
- "mi": Mutual information (MI). MI provides a general measure of dependencies between variables, in particular, positive, negative and nonlinear correlations. The caclulation of MI is implemented via applying adaptive partitioning method for deriving equal-probability bins (i.e., each bin contains approximately the same number of data points). The number of bins is

heuristically determined (the lower bound): $1 + log2(n)$, where n is the length of the vector. Because MI increases with entropy, we normalize it to allow comparison of different pairwise clone similarities: $2 * MI/[H(x) + H(y)]$, where $H(x)$ and $H(y)$ stand for the entropy for the vector x and y, respectively

See Also

sDmatCluster

Examples

```
# 1) generate an iid normal random matrix of 100x10
data <- matrix( rnorm(100*10,mean=0,sd=1), nrow=100, ncol=10)

# 2) calculate distance matrix using different metric
sMap <- sPipeline(data=data)
# 2a) using "pearson" metric
dist <- sDistance(data=data, metric="pearson")
# 2b) using "cos" metric
# dist <- sDistance(data=data, metric="cos")
# 2c) using "spearman" metric
# dist <- sDistance(data=data, metric="spearman")
# 2d) using "kendall" metric
# dist <- sDistance(data=data, metric="kendall")
# 2e) using "euclidean" metric
# dist <- sDistance(data=data, metric="euclidean")
# 2f) using "manhattan" metric
# dist <- sDistance(data=data, metric="manhattan")
# 2g) using "mi" metric
# dist <- sDistance(data=data, metric="mi")
```

---

sDmat — *Function to calculate distance matrix in high-dimensional input space but according to neighborhood relationships in 2D output space*

Description

sDmat is supposed to calculate distance (measured in high-dimensional input space) to neighbors (defined by based on 2D output space) for each of hexagons/rectangles

Usage

```
sDmat(sMap, which_neigh = 1,
  distMeasure = c("median", "mean", "min", "max"))
```

Arguments

sMap	an object of class "sMap"
which_neigh	which neighbors in 2D output space are used for the calculation. By default, it sets to "1" for direct neighbors, and "2" for neighbors within neighbors no more than 2, and so on
distMeasure	distance measure used to calculate distances in high-dimensional input space

Value

`dMat` a vector with the length of nHex. It stores the distance a hexaon/rectangle is away from its output-space-defined neighbors in high-dimensional input space

Note

"which_neigh" is defined in output 2D space, but "distMeasure" is defined in high-dimensional input space

See Also

sNeighAny

Examples

```
# 1) generate an iid normal random matrix of 100x10
data <- matrix( rnorm(100*10,mean=0,sd=1), nrow=100, ncol=10)

# 2) get trained using by default setup
sMap <- sPipeline(data=data)

# 3) calculate "median" distances in INPUT space to different neighbors in 2D OUTPUT space
# 3a) using direct neighbors in 2D OUTPUT space
dMat <- sDmat(sMap=sMap, which_neigh=1, distMeasure="median")
# 3b) using no more than 2-topological neighbors in 2D OUTPUT space
# dMat <- sDmat(sMap=sMap, which_neigh=2, distMeasure="median")
```

---

sDmatCluster *Function to partition a grid map into clusters*

Description

`sDmatCluster` is supposed to obtain clusters from a grid map. It returns an object of class "sBase".

Usage

```
sDmatCluster(sMap, which_neigh = 1,
  distMeasure = c("median", "mean", "min", "max"),
  clusterLinkage = c("average", "complete", "single", "bmh"))
```

Arguments

`sMap`	an object of class "sMap"
`which_neigh`	which neighbors in 2D output space are used for the calculation. By default, it sets to "1" for direct neighbors, and "2" for neighbors within neighbors no more than 2, and so on
`distMeasure`	distance measure used to calculate distances in high-dimensional input space. It can be one of "median", "mean", "min" and "max" measures
`clusterLinkage`	cluster linkage used to derive clusters. It can be "bmh", which accumulates a cluster just based on best-matching hexagons/rectanges but can not ensure each cluster is continuous. Instead, each cluster is continuous when using region-growing algorithm with one of "average", "complete" and "single" linkages

Value

an object of class "sBase", a list with following components:

- `seeds` — the vector to store cluster seeds, i.e., a list of local minima (in 2D output space) of distance matrix (in input space). They are represented by the indexes of hexagons/rectangles
- `bases` — the vector with the length of nHex to store the cluster memberships/bases, where nHex is the total number of hexagons/rectanges in the grid
- `call` — the call that produced this result

Note

The first item in the return "seeds" is the first cluster, whose memberships are those in the return "bases" that equals 1. The same relationship is held for the second item, and so on

See Also

`sPipeline`, `sDmatMinima`, `sBMH`, `sNeighDirect`, `sDistance`, `visDmatCluster`

Examples

```
# 1) generate an iid normal random matrix of 100x10
data <- matrix( rnorm(100*10,mean=0,sd=1), nrow=100, ncol=10)

# 2) get trained using by default setup
sMap <- sPipeline(data=data)

# 3) partition the grid map into clusters based on different criteria
# 3a) based on "bmh" criterion
# sBase <- sDmatCluster(sMap=sMap, which_neigh=1, distMeasure="median", clusterLinkage="bmh")
# 3b) using region-growing algorithm with linkage "average"
sBase <- sDmatCluster(sMap=sMap, which_neigh=1, distMeasure="median", clusterLinkage="average")

# 4) visualise clusters/bases partitioned from the sMap
visDmatCluster(sMap,sBase)
```

`sDmatMinima` *Function to identify local minima (in 2D output space) of distance matrix (in high-dimensional input space)*

Description

`sDmatMinima` is supposed to identify local minima of distance matrix (resulting from `sDmat`). The criterion of being local minima is that the distance associated with a hexagon/rectangle is always smaller than its direct neighbors (i.e., 1-neighborhood)

Usage

```
sDmatMinima(sMap, which_neigh = 1,
  distMeasure = c("median", "mean", "min", "max"))
```

Arguments

sMap	an object of class "sMap"
which_neigh	which neighbors in 2D output space are used for the calculation. By default, it sets to "1" for direct neighbors, and "2" for neighbors within neighbors no more than 2, and so on
distMeasure	distance measure used to calculate distances in high-dimensional input space. It can be one of "median", "mean", "min" and "max" measures

Value

minima	a vector to store a list of local minima (represented by the indexes of hexagons/rectangles

Note

Do not get confused by "which_neigh" and the criteria of being local minima. Both of them deal with 2D output space. However, "which_neigh" is used to assist in the calculation of distance matrix (so can be 1-neighborhood or more); instead, the criterion of being local minima is only 1-neighborhood in the strictest sense

See Also

sDmat, sNeighAny

Examples

```
# 1) generate an iid normal random matrix of 100x10
data <- matrix( rnorm(100*10,mean=0,sd=1), nrow=100, ncol=10)

# 2) get trained using by default setup
sMap <- sPipeline(data=data)

# 3) identify local minima of distance matrix based on "median" distances and direct neighbors
minima <- sDmatMinima(sMap=sMap, which_neigh=1, distMeasure="median")
```

---

sHexDist — *Function to calculate distances between hexagons/rectangles in a 2D grid*

Description

sHexDist is supposed to calculate euclidian distances between each pair of hexagons/rectangles in a 2D grid of input "sTopol" or "sMap" object. It returns a symmetric matrix containing pairwise distances.

Usage

```
sHexDist(sObj)
```

Arguments

sObj	an object of class "sTopol" or "sInit" or "sMap"

Value

dist	a symmetric matrix of nHex x nHex, containing pairwise distances, where nHex is the total number of hexagons/rectanges in the grid

Note

The return matrix has rows/columns ordered in the same order as the "coord" matrix of the input object does.

See Also

sTopology, sInitial

Examples

```
# 1) generate an iid normal random matrix of 100x10
data <- matrix( rnorm(100*10,mean=0,sd=1), nrow=100, ncol=10)

# 2) from this input matrix, determine nHex=5*sqrt(nrow(data))=50,
# but it returns nHex=61, via "sHexGrid(nHex=50)", to make sure a supra-hexagonal grid
sTopol <- sTopology(data=data, lattice="hexa", shape="suprahex")

# 3) initialise the codebook matrix using "uniform" method
sI <- sInitial(data=data, sTopol=sTopol, init="uniform")

# 4) calculate distances between hexagons/rectangles in a 2D grid based on different objects
# 4a) based on an object of class "sTopol"
dist <- sHexDist(sObj=sTopol)
# 4b) based on an object of class "sMap"
dist <- sHexDist(sObj=sI)
```

---

sHexGrid *Function to define a supra-hexagonal grid*

Description

sHexGrid is supposed to define a supra-hexagonal map grid. A supra-hexagon is a giant hexagon, which seamlessly consists of smaller hexagons. Due to the symmetric nature, it can be uniquely determined by specifying the radius away from the grid centroid. This function takes input the grid radius (or the number of hexagons in the grid, but will be adjusted to meet the definition of supra-hexagon), and returns a list (see 'Value' below) containing: the grid radius, the total number of hexagons in the grid, the 2D coordinates of the grid centroid, the step for each hexogan away from the grid centroid, and the 2D coordinates of all hexagons in the grid.

Usage

```
sHexGrid(r = NULL, nHex = NULL)
```

Arguments

r	an integer specifying the radius in a supra-hexagonal grid
nHex	the number of input hexagons in the grid

Value

a list with following components:

r	the grid radius
nHex	the total number of hexagons in the grid. It may differ from the input value; actually it is always no less than the input one to ensure a supra-hexagonal grid exactly formed
centroid	the 2D coordinates of the grid centroid
stepCentroid	a vector with the length of nHex. It stores how many steps a hexagon is awawy from the grid centroid ('1' for the centroid itself). Starting with the centroid, it orders outward. Also, for those hexagons of the same step, it orders from the rightmost in an anti-clock wise
coord	a matrix of nHex x 2 with each row specifying the 2D coordinates of a hexagon in the grid. The order of rows is the same as 'centroid' above
call	the call that produced this result

Note

The relationships among return values:

- $nHex = 1 + 6 * r * (r - 1)/2$
- $centroid = coord[1,]$
- $stepCentroid[1] = 1$
- $stepCentroid[2 : nHex] = unlist(sapply(2 : r, function(x)(c((1 + 6 * x * (x - 1)/2 - 6 * (x - 1) + 1) : (1 + 6 * x * (x - 1)/2)) >= 1) * x))$

Author(s)

Hai Fang <hfang@cs.bris.ac.uk>

References

http://supfam.org/SUPERFAMILY/dcGO/supraHex.html

See Also

sTopology

Examples

```
# The supra-hexagonal grid is exactly determined by specifying the radius.
res <- sHexGrid(r=2)

# The grid is determined according to the number of input hexagons (after being adjusted).
# The return res$nHex is always no less than the input one.
# It ensures a supra-hexagonal grid is exactly formed.
res <- sHexGrid(nHex=12)

# Ignore input nHex if r is also given
res <- sHexGrid(r=3, nHex=100)

# By default, r=3 if no parameters are specified
res <- sHexGrid()
```

sInitial *Function to initialise a sInit object given a topology and input data*

Description

`sInitial` is supposed to initialise an object of class "sInit" given a topology and input data. As a matter of fact, it initialises the codebook matrix (in input high-dimensional space). The return object inherits the topology information (i.e., a "sTopol" object from `sTopology`), along with initialised codebook matrix and method used.

Usage

```
sInitial(data, sTopol,
  init = c("linear", "uniform", "sample"))
```

Arguments

`data`	a data frame or matrix of input data
`sTopol`	an object of class "sTopol" (see `sTopology`)
`init`	an initialisation method. It can be one of "uniform", "sample" and "linear" initialisation methods

Value

an object of class "sInit", a list with following components:

`nHex`	the total number of hexagons/rectanges in the grid
`xdim`	x-dimension of the grid
`ydim`	y-dimension of the grid
`lattice`	the grid lattice
`shape`	the grid shape
`coord`	a matrix of nHex x 2, with each row corresponding to the coordinates of a hexagon/rectangle in the 2D map grid
`init`	an initialisation method
`codebook`	a codebook matrix of nHex x ncol(data), with each row corresponding to a prototype vector in input high-dimensional space
`call`	the call that produced this result

Note

The initialisation methods include:

- "uniform": the codebook matrix is uniformly initialised via randomly taking any values within the interval [min, max] of each column of input data
- "sample": the codebook matrix is initialised via randomly sampling/selecting input data
- "linear": the codebook matrix is linearly initialised along the first two greatest eigenvectors of input data

See Also

sTopology

Examples

```
# 1) generate an iid normal random matrix of 100x10
data <- matrix( rnorm(100*10,mean=0,sd=1), nrow=100, ncol=10)

# 2) from this input matrix, determine nHex=5*sqrt(nrow(data))=50,
# but it returns nHex=61, via "sHexGrid(nHex=50)", to make sure a supra-hexagonal grid
sTopol <- sTopology(data=data, lattice="hexa", shape="suprahex")

# 3) initialise the codebook matrix using different mehtods
# 3a) using "uniform" method
sI_uniform <- sInitial(data=data, sTopol=sTopol, init="uniform")
# 3b) using "sample" method
# sI_sample <- sInitial(data=data, sTopol=sTopol, init="sample")
# 3c) using "linear" method
# sI_linear <- sInitial(data=data, sTopol=sTopol, init="linear")
```

---

sMapOverlay — *Function to overlay additional data onto the trained map for viewing the distribution of that additional data*

Description

sMapOverlay is supposed to overlay additional data onto the trained map for viewing the distribution of that additional data. It returns an object of class "sMap". It is realised by first estimating the hit histogram weighted by the neighborhood kernel, and then calculating the distribution of the additional data over the map (similarly weighted by the neighborhood kernel). The final overlaid distribution of additional data is normalised by the hit histogram.

Usage

```
sMapOverlay(sMap, data, additional)
```

Arguments

sMap	an object of class "sMap"
data	a data frame or matrix of input data
additional	a numeric vector or numeric matrix used to overlay onto the trained map. It must have the length (if being vector) or row number (if matrix) being equal to the number of rows in input data

Value

an object of class "sMap", a list with following components:

nHex	the total number of hexagons/rectanges in the grid
xdim	x-dimension of the grid
ydim	y-dimension of the grid

lattice	the grid lattice
shape	the grid shape
coord	a matrix of nHex x 2, with rows corresponding to the coordinates of all hexagons/rectangles in the 2D map grid
init	an initialisation method
neighKernel	the training neighborhood kernel
codebook	a codebook matrix of nHex x ncol(additional), with rows corresponding to overlaid vectors
hits	a vector of nHex, each element meaning that a hexagon/rectangle contains the number of input data vectors being hit wherein
mqe	the mean quantization error for the "best" BMH
call	the call that produced this result

Note

Weighting by neighbor kernel is to avoid rigid overlaying by only focusing on the best-matching map nodes as there may exist several closest best-matching nodes for an input data vector.

See Also

`sPipeline`, `sBMH`, `sHexDist`, `visHexMulComp`

Examples

```
# 1) generate an iid normal random matrix of 100x10
data <- matrix( rnorm(100*10,mean=0,sd=1), nrow=100, ncol=10)
colnames(data) <- paste(rep(S,10), seq(1:10), sep="")

# 2) get trained using by default setup
sMap <- sPipeline(data=data)

# 3) overlay additional data onto the trained map
# here using the first two columns of the input "data" as "additional"
# codebook in "sOverlay" is the same as the first two columns of codebook in "sMap"
sOverlay <- sMapOverlay(sMap=sMap, data=data, additional=data[,1:2])

# 4) viewing the distribution of that additional data
visHexMulComp(sOverlay)
```

---

sNeighAny — *Function to calculate any neighbors for each hexagon/rectangle in a grid*

Description

sNeighAny is supposed to calculate any neighbors for each hexagon/rectangle in a regular 2D grid. It returns a matrix with rows for the self, and columns for its any neighbors.

Usage

```
sNeighAny(sObj)
```

Arguments

sObj — an object of class "sTopol" or "sInit" or "sMap"

Value

aNeigh — a matrix of nHex x nHex, containing distance info in terms of any neighbors, where nHex is the total number of hexagons/rectanges in the grid

Note

The return matrix has rows for the self, and columns for its neighbors. The non-zeros mean the distance away from its neighbors, and the zeros for the self-self. It has rows/columns ordered in the same order as the "coord" matrix of the input object does.

See Also

sNeighDirect

Examples

```
# 1) generate an iid normal random matrix of 100x10
data <- matrix( rnorm(100*10,mean=0,sd=1), nrow=100, ncol=10)

# 2) from this input matrix, determine nHex=5*sqrt(nrow(data))=50,
# but it returns nHex=61, via "sHexGrid(nHex=50)", to make sure a supra-hexagonal grid
sTopol <- sTopology(data=data, lattice="hexa", shape="suprahex")

# 3) initialise the codebook matrix using "uniform" method
sI <- sInitial(data=data, sTopol=sTopol, init="uniform")

# 4) calculate any neighbors based on different objects
# 4a) based on an object of class "sTopol"
aNeigh <- sNeighAny(sObj=sTopol)
# 4b) based on an object of class "sMap"
# aNeigh <- sNeighAny(sObj=sI)
```

---

sNeighDirect — *Function to calculate direct neighbors for each hexagon/rectangle in a grid*

Description

sNeighDirect is supposed to calculate direct neighbors for each hexagon/rectangle in a regular 2D grid. It returns a matrix with rows for the self, and columns for its direct neighbors.

Usage

```
sNeighDirect(sObj)
```

Arguments

sObj — an object of class "sTopol" or "sInit" or "sMap"

Value

dNeigh — a matrix of nHex x nHex, containing presence/absence info in terms of direct neighbors, where nHex is the total number of hexagons/rectanges in the grid

Note

The return matrix has rows for the self, and columns for its direct neighbors. The "1" means the presence of direct neighbors, "0" for the absence. It has rows/columns ordered in the same order as the "coord" matrix of the input object does.

See Also

sHexDist

Examples

```
# 1) generate an iid normal random matrix of 100x10
data <- matrix( rnorm(100*10,mean=0,sd=1), nrow=100, ncol=10)

# 2) from this input matrix, determine nHex=5*sqrt(nrow(data))=50,
# but it returns nHex=61, via "sHexGrid(nHex=50)", to make sure a supra-hexagonal grid
sTopol <- sTopology(data=data, lattice="hexa", shape="suprahex")

# 3) initialise the codebook matrix using "uniform" method
sI <- sInitial(data=data, sTopol=sTopol, init="uniform")

# 4) calculate direct neighbors based on different objects
# 4a) based on an object of class "sTopol"
dNeigh <- sNeighDirect(sObj=sTopol)
# 4b) based on an object of class "sMap"
# dNeigh <- sNeighDirect(sObj=sI)
```

---

sPipeline — *Function to setup the pipeline for completing ab initio training given the input data*

Description

sPipeline is supposed to finish ab inito training for the input data. It returns an object of class "sMap".

Usage

```
sPipeline(data = NULL, xdim = NULL, ydim = NULL,
  nHex = NULL, lattice = c("hexa", "rect"),
  shape = c("suprahex", "sheet"),
  init = c("linear", "uniform", "sample"),
  algorithm = c("batch", "sequential"),
  alphaType = c("invert", "linear", "power"),
  neighKernel = c("gaussian", "bubble", "cutgaussian", "ep", "gamma"),
  finetuneSustain = F, verbose = T)
```

Arguments

`data`	a data frame or matrix of input data
`xdim`	an integer specifying x-dimension of the grid
`ydim`	an integer specifying y-dimension of the grid
`nHex`	the number of hexagons/rectangles in the grid
`lattice`	the grid lattice, either "hexa" for a hexagon or "rect" for a rectangle
`shape`	the grid shape, either "suprahex" for a supra-hexagonal grid or "sheet" for a hexagonal/rectangle sheet
`init`	an initialisation method. It can be one of "uniform", "sample" and "linear" initialisation methods
`algorithm`	the training algorithm. It can be one of "sequential" and "batch" algorithm
`alphaType`	the alpha type. It can be one of "invert", "linear" and "power" alpha types
`neighKernel`	the training neighborhood kernel. It can be one of "gaussian", "bubble", "cutgaussian", "ep" and "gamma" kernels
`finetuneSustain`	logical to indicate whether sustain the "finetune" training. If true, it will repeat the "finetune" stage until the mean quantization error does get worse. By default, it sets to true
`verbose`	logical to indicate whether the messages will be displayed in the screen. By default, it sets to false for no display

Value

an object of class "sMap", a list with following components:

`nHex`	the total number of hexagons/rectanges in the grid
`xdim`	x-dimension of the grid
`ydim`	y-dimension of the grid
`lattice`	the grid lattice
`shape`	the grid shape
`coord`	a matrix of nHex x 2, with rows corresponding to the coordinates of all hexagons/rectangles in the 2D map grid
`init`	an initialisation method
`neighKernel`	the training neighborhood kernel
`codebook`	a codebook matrix of nHex x ncol(data), with rows corresponding to prototype vectors in input high-dimensional space
`hits`	a vector of nHex, each element meaning that a hexagon/rectangle contains the number of input data vectors being hit wherein
`mqe`	the mean quantization error for the "best" BMH
`call`	the call that produced this result

Note

The pipeline sequentially consists of:

- i) sTopology used to define the topology of a grid (with "suprahex" shape by default) according to the input data;
- ii) sInitial used to initialise the codebook matrix given the pre-defined topology and the input data (by default using "uniform" initialisation method);
- iii) sTrainology and sTrainSeq used to get the grid map trained at both "rough" and "finetune" stages. If instructed, sustain the "finetune" training until the mean quantization error does get worse;
- iv) sBMH used to identify the best-matching hexagons/rectangles (BMH) for the input data, and these response data are appended to the resulting object of "sMap" class.

See Also

sTopology, sInitial, sTrainology, sTrainSeq, sTrainBatch, sBMH, visHexMulComp

Examples

```
# 1) generate an iid normal random matrix of 100x10
data <- matrix( rnorm(100*10,mean=0,sd=1), nrow=100, ncol=10)
colnames(data) <- paste(rep(S,10), seq(1:10), sep="")

# 2) get trained using by default setup but with different neighborhood kernels
# 2a) with "gaussian" kernel
sMap <- sPipeline(data=data, neighKernel="gaussian")
# 2b) with "bubble" kernel
# sMap <- sPipeline(data=data, neighKernel="bubble")
# 2c) with "cutgaussian" kernel
# sMap <- sPipeline(data=data, neighKernel="cutgaussian")
# 2d) with "ep" kernel
# sMap <- sPipeline(data=data, neighKernel="ep")
# 2e) with "gamma" kernel
# sMap <- sPipeline(data=data, neighKernel="gamma")

# 3) visualise multiple component planes of a supra-hexagonal grid
visHexMulComp(sMap, colormap="jet", ncolors=20, zlim=c(-1,1), gp=grid::gpar(cex=0.8))
```

---

sTopology *Function to define the topology of a map grid*

Description

sTopology is supposed to define the topology of a 2D map grid. The topological shape can be either a supra-hexagonal grid or a hexagonal/rectangle sheet. It returns an object of "sTopol" class, containing: the total number of hexagons/rectangles in the grid, the grid xy-dimensions, the grid lattice, the grid shape, and the 2D coordinates of all hexagons/rectangles in the grid. The 2D coordinates can be directly used to measure distances between any pair of lattice hexagons/rectangles.

Usage

```
sTopology(data = NULL, xdim = NULL, ydim = NULL,
  nHex = NULL, lattice = c("hexa", "rect"),
  shape = c("suprahex", "sheet"))
```

Arguments

`data`	a data frame or matrix of input data
`xdim`	an integer specifying x-dimension of the grid
`ydim`	an integer specifying y-dimension of the grid
`nHex`	the number of hexagons/rectangles in the grid
`lattice`	the grid lattice, either "hexa" for a hexagon or "rect" for a rectangle
`shape`	the grid shape, either "suprahex" for a supra-hexagonal grid or "sheet" for a hexagonal/rectangle sheet

Value

an object of class "sTopol", a list with following components:

`nHex`	the total number of hexagons/rectanges in the grid. It is not always the same as the input nHex (if any); see "Note" below for the explaination
`xdim`	x-dimension of the grid
`ydim`	y-dimension of the grid
`lattice`	the grid lattice
`shape`	the grid shape
`coord`	a matrix of nHex x 2, with each row corresponding to the coordinates of a hexagon/rectangle in the 2D map grid
`call`	the call that produced this result

Note

The output of nHex depends on the input arguments and grid shape:

- How the input parameters are used to determine nHex is taken priority in the following order: "xdim & ydim" > "nHex" > "data"
- If both of xdim and ydim are given, $nHex = xdim * ydim$ for the "sheet" shape, $r = (min(xdim, ydim) + 1)/2$ for the "suprahex" shape
- If only data is input, $nHex = 5 * sqrt(dlen)$, where dlen is the number of rows of the input data
- With nHex in hand, it depends on the grid shape:
  - For "sheet" shape, xy-dimensions of sheet grid is determined according to the square root of the two biggest eigenvalues of the input data
  - For "suprahex" shape, see `sHexGrid` for calculating the grid radius r. The xdim (and ydim) is related to r via $xdim = 2 * r - 1$

See Also

`sHexGrid`, `visHexMapping`

Examples

```
# For "suprahex" shape
sTopol <- sTopology(xdim=3, ydim=3, lattice="hexa", shape="suprahex")

# Error: "The suprahex shape grid only allows for hexagonal lattice"
# sTopol <- sTopology(xdim=3, ydim=3, lattice="rect", shape="suprahex")

# For "sheet" shape with hexagonal lattice
sTopol <- sTopology(xdim=3, ydim=3, lattice="hexa", shape="sheet")

# For "sheet" shape with rectangle lattice
sTopol <- sTopology(xdim=3, ydim=3, lattice="rect", shape="sheet")

# By default, nHex=19 (i.e., r=3; xdim=ydim=5) for "suprahex" shape
sTopol <- sTopology(shape="suprahex")

# By default, xdim=ydim=5 (i.e., nHex=25) for "sheet" shape
sTopol <- sTopology(shape="sheet")

# Determine the topology of a supra-hexagonal grid based on input data
# 1) generate an iid normal random matrix of 100x10
data <- matrix(rnorm(100*10,mean=0,sd=1), nrow=100, ncol=10)
# 2) from this input matrix, determine nHex=5*sqrt(nrow(data))=50,
# but it returns nHex=61, via "sHexGrid(nHex=50)", to make sure a supra-hexagonal grid
sTopol <- sTopology(data=data, lattice="hexa", shape="suprahex")

# visualise a supre-hexagonal grid
visHexMapping(sTopol,mappingType="indexes")
```

---

sTrainBatch — *Function to implement training via batch algorithm*

Description

sTrainBatch is supposed to perform batch training algorithm. It requires three inputs: a "sMap" or "sInit" object, input data, and a "sTrain" object specifying training environment. The training is implemented iteratively, but instead of choosing a single input vector, the whole input matrix is used. In each training cycle, the whole input matrix first land in the map through identifying the corresponding winner hexagon/rectangle (BMH), and then the codebook matrix is updated via updating formula (see "Note" below for details). It returns an object of class "sMap".

Usage

```
sTrainBatch(sMap, data, sTrain)
```

Arguments

sMap	an object of class "sMap" or "sInit"
data	a data frame or matrix of input data
sTrain	an object of class "sTrain"

Value

an object of class "sMap", a list with following components:

`nHex`	the total number of hexagons/rectanges in the grid
`xdim`	x-dimension of the grid
`ydim`	y-dimension of the grid
`lattice`	the grid lattice
`shape`	the grid shape
`coord`	a matrix of nHex x 2, with each row corresponding to the coordinates of a hexagon/rectangle in the 2D map grid
`init`	an initialisation method
`neighKernel`	the training neighborhood kernel
`codebook`	a codebook matrix of nHex x ncol(data), with each row corresponding to a prototype vector in input high-dimensional space
`call`	the call that produced this result

Note

Updating formula is: $m_i(t+1) = \frac{\sum_{j=1}^{dlen} h_{wi}(t)x_j}{\sum_{j=1}^{dlen} h_{wi}(t)}$, where

- t denotes the training time/step
- x_j is an input vector j from the input data matrix (with $dlen$ rows in total)
- i and w stand for the hexagon/rectangle i and the winner BMH w, respectively
- $m_i(t+1)$ is the prototype vector of the hexagon i at time $t+1$
- $h_{wi}(t)$ is the neighborhood kernel, a non-increasing function of i) the distance d_{wi} between the hexagon/rectangle i and the winner BMH w, and ii) the radius δ_t at time t. There are five kernels available:
  - For "gaussian" kernel, $h_{wi}(t) = e^{-d_{wi}^2/(2*\delta_t^2)}$
  - For "cutguassian" kernel, $h_{wi}(t) = e^{-d_{wi}^2/(2*\delta_t^2)} * (d_{wi} \le \delta_t)$
  - For "bubble" kernel, $h_{wi}(t) = (d_{wi} \le \delta_t)$
  - For "ep" kernel, $h_{wi}(t) = (1 - d_{wi}^2/\delta_t^2) * (d_{wi} \le \delta_t)$
  - For "gamma" kernel, $h_{wi}(t) = 1/\Gamma(d_{wi}^2/(4 * \delta_t^2) + 2)$

See Also

sTrainology, visKernels

Examples

```
# 1) generate an iid normal random matrix of 100x10
data <- matrix( rnorm(100*10,mean=0,sd=1), nrow=100, ncol=10)

# 2) from this input matrix, determine nHex=5*sqrt(nrow(data))=50,
# but it returns nHex=61, via "sHexGrid(nHex=50)", to make sure a supra-hexagonal grid
sTopol <- sTopology(data=data, lattice="hexa", shape="suprahex")

# 3) initialise the codebook matrix using "uniform" method
sI <- sInitial(data=data, sTopol=sTopol, init="uniform")
```

```
# 4) define trainology at "rough" stage
sT_rough <- sTrainology(sMap=sI, data=data, stage="rough")

# 5) training at "rough" stage
sM_rough <- sTrainBatch(sMap=sI, data=data, sTrain=sT_rough)

# 6) define trainology at "finetune" stage
sT_finetune <- sTrainology(sMap=sI, data=data, stage="finetune")

# 7) training at "finetune" stage
sM_finetune <- sTrainBatch(sMap=sM_rough, data=data, sTrain=sT_rough)
```

`sTrainology` *Function to define trainology (training environment)*

Description

`sTrainology` is supposed to define the train-ology (i.e., the training environment/parameters). The trainology here refers to the training algorithm, the training stage, the stage-specific parameters (alpha type, initial alpha, initial radius, final radius and train length), and the training neighbor kernel used. It returns an object of class "sTrain".

Usage

```
sTrainology(sMap, data,
  algorithm = c("batch", "sequential"),
  stage = c("rough", "finetune", "complete"),
  alphaType = c("invert", "linear", "power"),
  neighKernel = c("gaussian", "bubble", "cutgaussian", "ep", "gamma"))
```

Arguments

`sMap`	an object of class "sMap" or "sInit"
`data`	a data frame or matrix of input data
`algorithm`	the training algorithm. It can be one of "sequential" and "batch" algorithm
`stage`	the training stage. The training can be achieved using two stages (i.e., "rough" and "finetune") or one stage only (i.e., "complete")
`alphaType`	the alpha type. It can be one of "invert", "linear" and "power" alpha types
`neighKernel`	the training neighbor kernel. It can be one of "gaussian", "bubble", "cutgaussian", "ep" and "gamma" kernels

Value

an object of class "sTrain", a list with following components:

`algorithm`	the training algorithm
`stage`	the training stage
`alphaType`	the alpha type
`alphaInitial`	the initial alpha

`radiusInitial`	the initial radius
`radiusFinal`	the final radius
`neighKernel`	the neighbor kernel
`call`	the call that produced this result

Note

Training stage-specific parameters:

- "radiusInitial": it depends on the grid shape and training stage
  - For "sheet" shape: it equals $max(1, ceiling(max(xdim, ydim)/8))$ at "rough" or "complete" stage, and $max(1, ceiling(max(xdim, ydim)/32))$ at "finetune" stage
  - For "suprahex" shape: it equals $max(1, ceiling(r/2))$ at "rough" or "complete" stage, and $max(1, ceiling(r/8))$ at "finetune" stage
- "radiusFinal": it depends on the training stage
  - At "rough" stage, it equals $radiusInitial/4$
  - At "finetune" or "complete" stage, it equals 1
- "trainLength": how many times the whole input data are set for training. It depends on the training stage and training algorithm
  - At "rough" stage, it equals $max(1, 10 * trainDepth)$
  - At "finetune" stage, it equals $max(1, 40 * trainDepth)$
  - At "complete" stage, it equals $max(1, 50 * trainDepth)$
  - When using "batch" algorithm and the trainLength equals 1 according to the above equation, the trainLength is forced to be 2 unless $radiusInitial$ equals $radiusFinal$
  - Where $trainDepth$ is the training depth, defined as $nHex/dlen$, i.e., how many hexagons/rectanges are used per the input data length (here $dlen$ refers to the number of rows)

See Also

sInitial

Examples

```
# 1) generate an iid normal random matrix of 100x10
data <- matrix( rnorm(100*10,mean=0,sd=1), nrow=100, ncol=10)

# 2) from this input matrix, determine nHex=5*sqrt(nrow(data))=50,
# but it returns nHex=61, via "sHexGrid(nHex=50)", to make sure a supra-hexagonal grid
sTopol <- sTopology(data=data, lattice="hexa", shape="suprahex")

# 3) initialise the codebook matrix using "uniform" method
sI <- sInitial(data=data, sTopol=sTopol, init="uniform")

# 4) define trainology at different stages
# 4a) define trainology at "rough" stage
sT_rough <- sTrainology(sMap=sI, data=data, stage="rough")
# 4b) define trainology at "finetune" stage
sT_finetune <- sTrainology(sMap=sI, data=data, stage="finetune")
# 4c) define trainology using "complete" stage
sT_complete <- sTrainology(sMap=sI, data=data, stage="complete")
```

sTrainSeq — *Function to implement training via sequential algorithm*

Description

sTrainSeq is supposed to perform sequential training algorithm. It requires three inputs: a "sMap" or "sInit" object, input data, and a "sTrain" object specifying training environment. The training is implemented iteratively, each training cycle consisting of: i) randomly choose one input vector; ii) determine the winner hexagon/rectangle (BMH) according to minimum distance of codebook matrix to the input vector; ii) update the codebook matrix of the BMH and its neighbors via updating formula (see "Note" below for details). It also returns an object of class "sMap".

Usage

```
sTrainSeq(sMap, data, sTrain)
```

Arguments

sMap	an object of class "sMap" or "sInit"
data	a data frame or matrix of input data
sTrain	an object of class "sTrain"

Value

an object of class "sMap", a list with following components:

nHex	the total number of hexagons/rectanges in the grid
xdim	x-dimension of the grid
ydim	y-dimension of the grid
lattice	the grid lattice
shape	the grid shape
coord	a matrix of nHex x 2, with each row corresponding to the coordinates of a hexagon/rectangle in the 2D map grid
init	an initialisation method
neighKernel	the training neighborhood kernel
codebook	a codebook matrix of nHex x ncol(data), with each row corresponding to a prototype vector in input high-dimensional space
call	the call that produced this result

Note

Updating formula is: $m_i(t+1) = m_i(t) + \alpha(t) * h_{wi}(t) * [x(t) - m_i(t)]$, where

- t denotes the training time/step
- i and w stand for the hexagon/rectangle i and the winner BMH w, respectively
- $x(t)$ is an input vector randomly choosen (from the input data) at time t
- $m_i(t)$ and $m_i(t+1)$ are respectively the prototype vectors of the hexagon i at time t and $t+1$

- $\alpha(t)$ is the learning rate at time t. There are three types of learning rate functions:
  - For "linear" function, $\alpha(t) = \alpha_0 * (1 - t/T)$
  - For "power" function, $\alpha(t) = \alpha_0 * (0.005/\alpha_0)^{t/T}$
  - For "invert" function, $\alpha(t) = \alpha_0/(1 + 100 * t/T)$
  - Where α_0 is the initial learing rate (typically, $\alpha_0 = 0.5$ at "rough" stage, $\alpha_0 = 0.05$ at "finetune" stage), T is the length of training time/step (often being set to input data length, i.e., the total number of rows)
- $h_{wi}(t)$ is the neighborhood kernel, a non-increasing function of i) the distance d_{wi} between the hexagon/rectangle i and the winner BMH w, and ii) the radius δ_t at time t. There are five kernels available:
  - For "gaussian" kernel, $h_{wi}(t) = e^{-d_{wi}^2/(2*\delta_t^2)}$
  - For "cutguassian" kernel, $h_{wi}(t) = e^{-d_{wi}^2/(2*\delta_t^2)} * (d_{wi} \le \delta_t)$
  - For "bubble" kernel, $h_{wi}(t) = (d_{wi} \le \delta_t)$
  - For "ep" kernel, $h_{wi}(t) = (1 - d_{wi}^2/\delta_t^2) * (d_{wi} \le \delta_t)$
  - For "gamma" kernel, $h_{wi}(t) = 1/\Gamma(d_{wi}^2/(4 * \delta_t^2) + 2)$

See Also

sTrainology, visKernels

Examples

```
# 1) generate an iid normal random matrix of 100x10
data <- matrix( rnorm(100*10,mean=0,sd=1), nrow=100, ncol=10)

# 2) from this input matrix, determine nHex=5*sqrt(nrow(data))=50,
# but it returns nHex=61, via "sHexGrid(nHex=50)", to make sure a supra-hexagonal grid
sTopol <- sTopology(data=data, lattice="hexa", shape="suprahex")

# 3) initialise the codebook matrix using "uniform" method
sI <- sInitial(data=data, sTopol=sTopol, init="uniform")

# 4) define trainology at "rough" stage
sT_rough <- sTrainology(sMap=sI, data=data, stage="rough")

# 5) training at "rough" stage
sM_rough <- sTrainSeq(sMap=sI, data=data, sTrain=sT_rough)

# 6) define trainology at "finetune" stage
sT_finetune <- sTrainology(sMap=sI, data=data, stage="finetune")

# 7) training at "finetune" stage
sM_finetune <- sTrainSeq(sMap=sM_rough, data=data, sTrain=sT_rough)
```

---

sWriteData — *Function to write out the best-matching hexagons and/or cluster bases in terms of data*

Description

sWriteData is supposed to write out the best-matching hexagons and/or cluster bases in terms of data.

Usage

```
sWriteData(sMap, data, sBase = NULL, filename = NULL,
  keep.data = F)
```

Arguments

sMap	an object of class "sMap" or a codebook matrix
data	a data frame or matrix of input data
sBase	an object of class "sBase"
filename	a character string naming a filename
keep.data	logical to indicate whether or not to also write out the input data. By default, it sets to false for not keeping it. It is highly expensive to keep the large data sets

Value

a data frame with following components:

ID	ID for data. It inherits the rownames of data (if exists). Otherwise, it is sequential integer values starting with 1 and ending with dlen, the total number of rows of the input data
Hexagon_index	the index for best-matching hexagons
Cluster_base	optional, it is only appended when sBase is given. It stores the cluster memberships/bases
data	optional, it is only appended when keep.data is true

Note

If "filename" is not NULL, a tab-delimited text file will be also written out. If "sBase" is not NULL and comes from the "sMap" partition, then cluster bases are also appended. if "keep.data" is true, the data will be part of output.

See Also

sBMH

Examples

```
# 1) generate an iid normal random matrix of 100x10
data <- matrix( rnorm(100*10,mean=0,sd=1), nrow=100, ncol=10)

# 2) get trained using by default setup
sMap <- sPipeline(data=data)

# 3) write datas BMH hitting the trained map
output <- sWriteData(sMap=sMap, data=data, filename="sData_output.txt")

# 4) partition the grid map into cluster bases
sBase <- sDmatCluster(sMap=sMap, which_neigh=1,
distMeasure="median", clusterLinkage="average")

# 5) write datas BMH and cluster bases
output <- sWriteData(sMap=sMap, data=data, sBase=sBase, filename="sData_base_output.txt")
```

visColorbar *Function to define a colorbar*

Description

visColorbar is supposed to define a colorbar

Usage

```
visColorbar(colormap = c("bwr", "jet", "gbr", "wyr", "br", "yr", "rainbow", "wb"),
  ncolors = 40, zlim = c(0, 1), gp = grid::gpar())
```

Arguments

colormap	short name for the colormap. It can be one of "jet" (jet colormap), "bwr" (blue-white-red colormap), "gbr" (green-black-red colormap), "wyr" (white-yellow-red colormap), "br" (black-red colormap), "yr" (yellow-red colormap), "wb" (white-black colormap), and "rainbow" (rainbow colormap, that is, red-yellow-green-cyan-blue-magenta). Alternatively, any hyphen-separated HTML color names, e.g. "blue-black-yellow", "royalblue-white-sandybrown", "darkgreen-white-darkviolet". A list of standard color names can be found in http://html-color-codes.info/color-names
ncolors	the number of colors specified
zlim	the minimum and maximum z values for which colors should be plotted, defaulting to the range of the finite values of z. Each of the given colors will be used to color an equispaced interval of this range. The midpoints of the intervals cover the range, so that values just outside the range will be plotted
gp	an object of class gpar, typically the output from a call to the function gpar (i.e., a list of graphical parameter settings)

Value

invisibly

Note

none

See Also

visColormap, visHexMulComp, visCompReorder

Examples

```
# draw "blue-white-red" colorbar
visColorbar(colormap="bwr")
```

visColormap — *Function to define a colormap*

Description

`visColormap` is supposed to define a colormap. It returns a function, which will take an integer argument specifying how many colors interpolate the given colormap.

Usage

```
visColormap(colormap = c("bwr", "jet", "gbr", "wyr", "br", "yr", "rainbow", "wb"))
```

Arguments

`colormap`	short name for the colormap

Value

`palette.name`	a function that takes an integer argument for generating that number of colors interpolating the given sequence

Note

The input colormap includes:

- "jet": jet colormap
- "bwr": blue-white-red
- "gbr": green-black-red
- "wyr": white-yellow-red
- "br": black-red
- "yr": yellow-red
- "wb": white-black
- "rainbow": rainbow colormap, that is, red-yellow-green-cyan-blue-magenta
- Alternatively, any hyphen-separated HTML color names, e.g. "blue-black-yellow", "royalblue-white-sandybrown", "darkblue-lightblue-lightyellow-darkorange", "darkgreen-white-darkviolet", "darkgreen-lightgreen-lightpink-darkred". A list of standard color names can be found in `http://html-color-codes.info/color-names`

See Also

`visHexComp`

Examples

```
# 1) define "blue-white-red" colormap
palette.name <- visColormap(colormap="bwr")

# 2) use the return function "palette.name" to generate 10 colors spanning "bwr"
palette.name(10)
```

visCompReorder — *Function to visualise multiple component planes reorded within a sheet-shape rectangle grid*

Description

visCompReorder is supposed to visualise multiple component planes reorded within a sheet-shape rectangle grid

Usage

```
visCompReorder(sMap, sReorder, margin = rep(0.1, 4),
  height = 7, title.rotate = 0, title.xy = c(0.45, 1),
  colormap = c("bwr", "jet", "gbr", "wyr", "br", "yr", "rainbow", "wb"),
  ncolors = 40, zlim = NULL,
  border.color = "transparent", gp = grid::gpar())
```

Arguments

sMap	an object of class "sMap"
sReorder	an object of class "sReorder"
margin	margins as units of length 4 or 1
height	a numeric value specifying the height of device
title.rotate	the rotation of the title
title.xy	the coordinates of the title
colormap	short name for the colormap. It can be one of "jet" (jet colormap), "bwr" (blue-white-red colormap), "gbr" (green-black-red colormap), "wyr" (white-yellow-red colormap), "br" (black-red colormap), "yr" (yellow-red colormap), "wb" (white-black colormap), and "rainbow" (rainbow colormap, that is, red-yellow-green-cyan-blue-magenta). Alternatively, any hyphen-separated HTML color names, e.g. "blue-black-yellow", "royalblue-white-sandybrown", "darkgreen-white-darkviolet". A list of standard color names can be found in http://html-color-codes.info/color-names
ncolors	the number of colors specified
zlim	the minimum and maximum z values for which colors should be plotted, defaulting to the range of the finite values of z. Each of the given colors will be used to color an equispaced interval of this range. The midpoints of the intervals cover the range, so that values just outside the range will be plotted
border.color	the border color for each hexagon
gp	an object of class "gpar". It is the output from a call to the function "gpar" (i.e., a list of graphical parameter settings)

Value

invisible

Note

none

See Also

visVp, visHexComp, visColorbar, sCompReorder

Examples

```
# 1) generate data with three different distributions, each with an iid normal random matrix of 1000 x 3
data <- cbind(matrix(rnorm(1000*3,mean=0,sd=1), nrow=1000, ncol=3),
matrix(rnorm(1000*3,mean=0.5,sd=1), nrow=1000, ncol=3),
matrix(rnorm(1000*3,mean=-0.5,sd=1), nrow=1000, ncol=3))
colnames(data) <- c("S1","S1","S1","S2","S2","S2","S3","S3","S3")

# 2) sMap resulted from using by default setup
sMap <- sPipeline(data=data)

# 3) reorder component planes
sReorder <- sCompReorder(sMap=sMap, amplifier=2, metric="none")

# 4) visualise multiple component planes reorded within a sheet-shape rectangle grid
visCompReorder(sMap=sMap, sReorder=sReorder, margin=rep(0.1,4), height=7,
title.rotate=0, title.xy=c(0.45, 1), colormap="gbr", ncolors=10, zlim=c(-1,1),
border.color="transparent")
```

---

visDmatCluster — *Function to visualise clusters/bases partitioned from a supra-hexagonal grid*

Description

visDmatCluster is supposed to visualise clusters/bases partitioned from a supra-hexagonal grid

Usage

```
visDmatCluster(sMap, sBase, height = 7,
  margin = rep(0.1, 4), area.size = 1,
  gp = grid::gpar(cex = 0.8, font = 2, col.label = "black"),
  border.color = "transparent",
  colormap = c("rainbow", "jet", "bwr", "gbr", "wyr", "br", "yr", "wb"),
  clip = c("on", "inherit", "off"), newpage = T)
```

Arguments

sMap	an object of class "sMap"
sBase	an object of class "sBase"
height	a numeric value specifying the height of device
margin	margins as units of length 4 or 1
area.size	an inteter or a vector specifying the area size of each hexagon
gp	an object of class "gpar". It is the output from a call to the function "gpar" (i.e., a list of graphical parameter settings)
border.color	the border color for each hexagon

`colormap`	short name for the colormap. It can be one of "jet" (jet colormap), "bwr" (blue-white-red colormap), "gbr" (green-black-red colormap), "wyr" (white-yellow-red colormap), "br" (black-red colormap), "yr" (yellow-red colormap), "wb" (white-black colormap), and "rainbow" (rainbow colormap, that is, red-yellow-green-cyan-blue-magenta). Alternatively, any hyphen-separated HTML color names, e.g. "blue-black-yellow", "royalblue-white-sandybrown", "darkgreen-white-darkviolet". A list of standard color names can be found in `http://html-color-codes.info/color-names`
`clip`	either "on" for clipping to the extent of this viewport, "inherit" for inheriting the clipping region from the parent viewport, or "off" to turn clipping off altogether
`newpage`	logical to indicate whether to open a new page. By default, it sets to true for opening a new page

Value

invisible

Note

none

See Also

`sDmatCluster`, `visColormap`, `visHexGrid`

Examples

```
# 1) generate an iid normal random matrix of 100x10
data <- matrix( rnorm(100*10,mean=0,sd=1), nrow=100, ncol=10)

# 2) get trained using by default setup
sMap <- sPipeline(data=data)

# 3) partition the grid map into clusters using region-growing algorithm
sBase <- sDmatCluster(sMap=sMap, which_neigh=1,
distMeasure="median", clusterLinkage="average")

# 4) visualise clusters/bases partitioned from the sMap
visDmatCluster(sMap,sBase)
```

`visHexComp` *Function to visualise a component plane of a supra-hexagonal grid*

Description

`visHexComp` is supposed to visualise a supra-hexagonal grid in the context of viewport

Usage

```
visHexComp(sMap, comp, margin = rep(0.6, 4),
  area.size = 1,
  colormap = c("bwr", "jet", "gbr", "wyr", "br", "yr", "rainbow", "wb"),
  ncolors = 40, zlim = c(0, 1),
  border.color = "transparent", newpage = T)
```

Arguments

sMap	an object of class "sMap"
comp	a component/column of codebook matrix from an object "sMap"
margin	margins as units of length 4 or 1
area.size	an inteter or a vector specifying the area size of each hexagon
colormap	short name for the colormap. It can be one of "jet" (jet colormap), "bwr" (blue-white-red colormap), "gbr" (green-black-red colormap), "wyr" (white-yellow-red colormap), "br" (black-red colormap), "yr" (yellow-red colormap), "wb" (white-black colormap), and "rainbow" (rainbow colormap, that is, red-yellow-green-cyan-blue-magenta). Alternatively, any hyphen-separated HTML color names, e.g. "blue-black-yellow", "royalblue-white-sandybrown", "darkgreen-white-darkviolet". A list of standard color names can be found in http://html-color-codes.info/color-names
ncolors	the number of colors specified
zlim	the minimum and maximum z values for which colors should be plotted, defaulting to the range of the finite values of z. Each of the given colors will be used to color an equispaced interval of this range. The midpoints of the intervals cover the range, so that values just outside the range will be plotted
border.color	the border color for each hexagon
newpage	a logical to indicate whether or not to open a new page

Value

invisible

Note

none

See Also

visColormap, visHexGrid

Examples

```
# 1) generate an iid normal random matrix of 100x10
data <- matrix( rnorm(100*10,mean=0,sd=1), nrow=100, ncol=10)
colnames(data) <- paste(rep(S,10), seq(1:10), sep="")

# 2) sMap resulted from using by default setup
sMap <- sPipeline(data=data)

# 3) visualise the first component plane with a supra-hexagonal grid
visHexComp(sMap, comp=sMap$codebook[,1], colormap="jet", ncolors=100, zlim=c(-1,1))
```

visHexGrid *Function to visualise a supra-hexagonal grid*

Description

visHexGrid is supposed to visualise a supra-hexagonal grid

Usage

```
visHexGrid(hbin, area.size = 1, border.color = NULL,
  fill.color = NULL)
```

Arguments

hbin	an object of class "hexbin"
area.size	an inteter or a vector specifying the area size of each hexagon
border.color	the border color for each hexagon
fill.color	the filled color for each hexagon

Value

invisible

Note

none

See Also

visHexComp

Examples

```
# 1) generate an iid normal random matrix of 100x10
data <- matrix( rnorm(100*10,mean=0,sd=1), nrow=100, ncol=10)
colnames(data) <- paste(rep(S,10), seq(1:10), sep="")

# 2) sMap resulted from using by default setup
sMap <- sPipeline(data=data)

# 3) create an object of "hexbin" class from sMap
dat <- data.frame(sMap$coord)
xdim <- sMap$xdim
ydim <- sMap$ydim
hbin <- hexbin::hexbin(dat$x, dat$y, xbins=xdim-1, shape=sqrt(0.75)*ydim/xdim)

# 4) visualise hbin object
vp <- hexbin::hexViewport(hbin)
visHexGrid(hbin)
```

`visHexMapping` *Function to visualise various mapping items within a supra-hexagonal grid*

Description

`visHexMapping` is supposed to visualise various mapping items within a supra-hexagonal grid

Usage

```
visHexMapping(sObj,
  mappingType = c("indexes", "hits", "dist", "antidist", "bases", "customized"),
  labels = NULL, height = 7, margin = rep(0.1, 4),
  area.size = 1,
  gp = grid::gpar(cex = 0.7, font = 1, col.label = "black"),
  border.color = "black", fill.color = "transparent",
  clip = c("on", "inherit", "off"), newpage = T)
```

Arguments

`sObj`	an object of class "sMap" or "sInit" or "sTopol"
`mappingType`	the mapping type, can be "indexes", "hits", "dist", "antidist", "bases", and "customized"
`labels`	NULL or a vector with the length of nHex
`height`	a numeric value specifying the height of device
`margin`	margins as units of length 4 or 1
`area.size`	an inteter or a vector specifying the area size of each hexagon
`gp`	an object of class "gpar". It is the output from a call to the function "gpar" (i.e., a list of graphical parameter settings)
`border.color`	the border color for each hexagon
`fill.color`	the filled color for each hexagon
`clip`	either "on" for clipping to the extent of this viewport, "inherit" for inheriting the clipping region from the parent viewport, or "off" to turn clipping off altogether
`newpage`	logical to indicate whether to open a new page. By default, it sets to true for opening a new page

Value

invisible

Note

The mappingType includes:

- "indexes": the index of hexagons in a supra-hexagonal grid
- "hits": the number of input data vectors hitting the hexagons
- "dist": distance (in high-dimensional input space) to neighbors (defined in 2D output space)
- "antidist": the oppose version of "dist"
- "bases": clusters partitioned from the sMap
- "customized": displaying input "labels"

See Also

sDmat, sDmatCluster, visHexGrid

Examples

```
# 1) generate data with three different distributions, each with an iid normal random matrix of 1000 x 3
data <- cbind(matrix(rnorm(1000*3,mean=0,sd=1), nrow=1000, ncol=3),
matrix(rnorm(1000*3,mean=0.5,sd=1), nrow=1000, ncol=3),
matrix(rnorm(1000*3,mean=-0.5,sd=1), nrow=1000, ncol=3))
colnames(data) <- c("S1","S1","S1","S2","S2","S2","S3","S3","S3")

# 2) sMap resulted from using by default setup
sMap <- sPipeline(data=data)

# 3) visualise supported mapping items within a supra-hexagonal grid
# 3a) for indexes of hexagons
visHexMapping(sMap,mappingType="indexes")
# 3b) for the number of input data vectors hitting the hexagons
visHexMapping(sMap,mappingType="hits")
# 3c) for distance (in high-dimensional input space) to neighbors (defined in 2D output space)
visHexMapping(sMap,mappingType="dist")
# 3d) for anti-distance (in high-dimensional input space) to neighbors (defined in 2D output space)
visHexMapping(sMap,mappingType="antidist")
# 3e) for clusters/bases partitioned from the sMap
visHexMapping(sMap,mappingType="bases")
```

---

visHexMulComp *Function to visualise multiple component planes of a supra-hexagonal grid*

Description

visHexMulComp is supposed to visualise multiple component planes of a supra-hexagonal grid

Usage

```
visHexMulComp(sMap, margin = rep(0.1, 4), height = 7,
  title.rotate = 0, title.xy = c(0.45, 1),
  colormap = c("bwr", "jet", "gbr", "wyr", "br", "yr", "rainbow", "wb"),
  ncolors = 40, zlim = NULL,
  border.color = "transparent", gp = grid::gpar())
```

Arguments

sMap	an object of class "sMap"
margin	margins as units of length 4 or 1
height	a numeric value specifying the height of device
title.rotate	the rotation of the title
title.xy	the coordinates of the title

colormap
: short name for the colormap. It can be one of "jet" (jet colormap), "bwr" (blue-white-red colormap), "gbr" (green-black-red colormap), "wyr" (white-yellow-red colormap), "br" (black-red colormap), "yr" (yellow-red colormap), "wb" (white-black colormap), and "rainbow" (rainbow colormap, that is, red-yellow-green-cyan-blue-magenta). Alternatively, any hyphen-separated HTML color names, e.g. "blue-black-yellow", "royalblue-white-sandybrown", "darkgreen-white-darkviolet". A list of standard color names can be found in http://html-color-codes.info/color-names

ncolors
: the number of colors specified

zlim
: the minimum and maximum z values for which colors should be plotted, defaulting to the range of the finite values of z. Each of the given colors will be used to color an equispaced interval of this range. The midpoints of the intervals cover the range, so that values just outside the range will be plotted

border.color
: the border color for each hexagon

gp
: an object of class gpar, typically the output from a call to the function gpar (i.e., a list of graphical parameter settings)

Value

invisible

Note

none

See Also

visVp, visHexComp, visColorbar

Examples

```
# 1) generate data with three different distributions, each with an iid normal random matrix of 1000 x 3
data <- cbind(matrix(rnorm(1000*3,mean=0,sd=1), nrow=1000, ncol=3),
matrix(rnorm(1000*3,mean=0.5,sd=1), nrow=1000, ncol=3),
matrix(rnorm(1000*3,mean=-0.5,sd=1), nrow=1000, ncol=3))
colnames(data) <- c("S1","S1","S1","S2","S2","S2","S3","S3","S3")

# 2) sMap resulted from using by default setup
sMap <- sPipeline(data=data)

# 3) visualise multiple component planes of a supra-hexagonal grid
visHexMulComp(sMap, colormap="jet", ncolors=20, zlim=c(-1,1), gp=grid::gpar(cex=0.8))
```

---

visHexPattern *Function to visualise codebook matrix or input patterns within a supra-hexagonal grid*

Description

visHexPattern is supposed to codebook matrix or input patterns within a supra-hexagonal grid.

Usage

```
visHexPattern(sObj,
  plotType = c("lines", "bars", "radars"),
  pattern = NULL, height = 7, margin = rep(0.1, 4),
  colormap = c("customized", "bwr", "jet", "gbr", "wyr", "br", "yr", "rainbow", "wb"),
  customized.color = "red", zeropattern.color = "gray",
  legend.cex = 0.8, newpage = T)
```

Arguments

- `sObj` an object of class "sMap" or "sTopol" or "sInit"
- `plotType` the plot type, can be "lines" for line/point graph, "bars" for bar graph, "radars" for radar graph
- `pattern` By default, it sets to "NULL" for the codebook matrix. It is intended for the user-input patterns, i.e., a matrix with the dimension of nHex x nPattern, where nHex is the number of hexagons and nPattern is the number of elements for each pattern
- `height` a numeric value specifying the height of device
- `margin` margins as units of length 4 or 1
- `colormap` short name for the predifined colormap, and "customized" for custom input (see the next 'customized.color'). The predifined colormap can be one of "jet" (jet colormap), "bwr" (blue-white-red colormap), "gbr" (green-black-red colormap), "wyr" (white-yellow-red colormap), "br" (black-red colormap), "yr" (yellow-red colormap), "wb" (white-black colormap), and "rainbow" (rainbow colormap, that is, red-yellow-green-cyan-blue-magenta). Alternatively, any hyphen-separated HTML color names, e.g. "blue-black-yellow", "royalblue-white-sandybrown", "darkgreen-white-darkviolet". A list of standard color names can be found in http://html-color-codes.info/color-names
- `customized.color` the customized color for pattern visualisation
- `zeropattern.color` the color for zero horizental line
- `legend.cex` a numerical value giving the amount by which legend text should be magnified relative to the default (i.e., 1)
- `newpage` logical to indicate whether to open a new page. By default, it sets to true for opening a new page

Value

invisible

Note

The "plotType" includes:

- "lines": line plot. If multple colors are given, the points are also plotted. When the pattern involves both positive and negative values, zero horizental line is also shown
- "bars": bar plot. When the pattern involves both positive and negative values, the zero horizental line is in the middle of the hexagon; otherwise at the top of the hexagon for all negative values, and at the bottom for all positive values

- "radars": radar plot. Each radar diagram represents one pattern, wherein each element value is proportional to the distance from the center. Note, it starts on the right and wind counterclockwise around the circle

See Also

sPipeline, visColormap

Examples

```
# 1) generate data with three different distributions, each with an iid normal random matrix of 1000 x 3
data <- cbind(matrix(rnorm(1000*3,mean=0,sd=1), nrow=1000, ncol=3),
matrix(rnorm(1000*3,mean=0.5,sd=1), nrow=1000, ncol=3),
matrix(rnorm(1000*3,mean=-0.5,sd=1), nrow=1000, ncol=3))
colnames(data) <- c("S1","S1","S1","S2","S2","S2","S3","S3","S3")

# 2) sMap resulted from using by default setup
sMap <- sPipeline(data=data)

# 3) plot codebook patterns using different types
# 3a) line plot
visHexPattern(sMap, plotType="lines", customized.color="red", zeropattern.color="gray")
# visHexPattern(sMap, plotType="lines", customized.color=rep(c("red","green","blue"),each=3))
# 3b) bar plot
visHexPattern(sMap, plotType="bars")
# visHexPattern(sMap, plotType="bars", colormap="jet", legend.cex=0.8)
# visHexPattern(sMap, plotType="bars", customized.color=rep(c("red","green","blue"),each=3))
# 3c) radar plot
visHexPattern(sMap, plotType="radars")
# visHexPattern(sMap, plotType="radars", colormap="jet", legend.cex=0.8)
# visHexPattern(sMap, plotType="radars", customized.color=rep(c("red","green","blue"),each=3))

# 4) plot user-input patterns using different types
# 4a) generate pattern data with two different groups "S" and "T"
nHex <- sMap$nHex
pattern <- cbind(matrix(runif(nHex*3,min=0,max=1), nrow=nHex, ncol=3),
matrix(runif(nHex*3,min=1,max=2), nrow=nHex, ncol=3))
colnames(pattern) <- c("S1","S2","S3","T1","T2","T3")
# 4b) for line plot
visHexPattern(sMap, plotType="lines", pattern=pattern, customized.color="red", zeropattern.color="gray")
# visHexPattern(sMap, plotType="lines", pattern=pattern, customized.color=rep(c("red","green"),each=3))
# 4c) for bar plot
visHexPattern(sMap, plotType="bars", pattern=pattern, customized.color=rep(c("red","green"),each=3))
# 4d) for radar plot
visHexPattern(sMap, plotType="radars", pattern=pattern, customized.color=rep(c("red","green"),each=3))
```

---

visKernels *Function to visualize neighborhood kernels*

Description

visKernels is supposed to visualize a series of neighborhood kernels, each of which is a non-increasing functions of: i) the distance d_{wi} between the hexagon/rectangle i and the winner w, and ii) the radius δ_t at time t.

Usage

```
visKernels(newpage = T)
```

Arguments

newpage	logical to indicate whether to open a new page. By default, it sets to true for opening a new page

Value

invisible

Note

There are five kernels that are currently supported:

- For "gaussian" kernel, $h_{wi}(t) = e^{-d_{wi}^2/(2*\delta_t^2)}$
- For "cutguassian" kernel, $h_{wi}(t) = e^{-d_{wi}^2/(2*\delta_t^2)} * (d_{wi} \le \delta_t)$
- For "bubble" kernel, $h_{wi}(t) = (d_{wi} \le \delta_t)$
- For "ep" kernel, $h_{wi}(t) = (1 - d_{wi}^2/\delta_t^2) * (d_{wi} \le \delta_t)$
- For "gamma" kernel, $h_{wi}(t) = 1/\Gamma(d_{wi}^2/(4 * \delta_t^2) + 2)$

These kernels above are displayed within a plot for each fixed radius. Three different radii (i.e., 1 and 2) are illustrated.

See Also

sTrainSeq, sTrainBatch

Examples

```
# visualise currently supported five kernels
visKernels()
```

---

visVp — *Function to create viewports for multiple supra-hexagonal grids*

Description

visVp is supposed to create viewports, which describe rectangular regions on a graphics device and define a number of coordinate systems for each of supra-hexagonal grids.

Usage

```
visVp(height = 7, xdim = 1, ydim = 1, colNum = 1,
  rowNum = 1, gp = grid::gpar())
```

Arguments

`height`	a numeric value specifying the height of device
`xdim`	an integer specifying x-dimension of the grid
`ydim`	an integer specifying y-dimension of the grid
`colNum`	an integer specifying the number of columns
`rowNum`	an integer specifying the number of rows
`gp`	an object of class gpar, typically the output from a call to the function gpar (i.e., a list of graphical parameter settings)

Value

`vpnames`	an R object of "viewport" class

Note

none

See Also

`visHexMulComp`, `visCompReorder`

Examples

```
# 1) create 5x5 viewports
vpnames <- visVp(colNum=5, rowNum=5)

# 2) look at names of these viewports
vpnames
```

`Xiang` *Arabidopsis embryo gene expression dataset from Xiang et al. (2011)*

Description

Arabidopsis embryo dataset contains gene expression levels (3625 genes and 7 embryo samples) from Xiang et al. (2011). This dataset has been pre-processed: capping into floor of intensity 777.6; 2-base logarithmic transformation; row/gene centering; and keeping genes with at least 2-fold changes (in any stage) as compared to the average over embryo stages.

Usage

```
data(Xiang)
```

Value

`Xiang`	a gene expression matrix of 3625 genes x 7 stage samples. These embryo stages are: zygote, quadrant, globular, heart, torpedo, bent, and mature.

References

Xiang et al. (2011) Genome-wide analysis reveals gene expression and metabolic network dynamics during embryo development in Arabidopsis. *Plant Physiol*, 156(1):346-356.

Index

∗Topic **datasets**
Fang, 2
Golub, 3
Xiang, 42

Fang, 2

Golub, 3

sBMH, 3, *6*, *10*, *16*, *20*, *28*
sCompReorder, 5, *32*
sDistance, *5*, *6*, 7, *10*
sDmat, 8, *10*, *11*, *37*
sDmatCluster, *8*, 9, *33*, *37*
sDmatMinima, *10*, 10
sHexDist, 11, *16*, *18*
sHexGrid, 12, *21*
sInitial, *12*, 14, *20*, *25*
sMapOverlay, 15
sNeighAny, *9*, *11*, 16
sNeighDirect, *10*, *17*, 17
sPipeline, *4*, *6*, *10*, *16*, 18, *40*
sTopology, *6*, *12*, *13*, *15*, *20*, 20
sTrainBatch, *20*, 22, *41*
sTrainology, *20*, *23*, 24, *27*
sTrainSeq, *20*, 26, *41*
sWriteData, 27

visColorbar, 29, *32*, *38*
visColormap, *29*, 30, *33*, *34*, *40*
visCompReorder, *6*, *29*, 31, *42*
visDmatCluster, *10*, 32
visHexComp, *30*, *32*, 33, *35*, *38*
visHexGrid, *33*, *34*, 35, *37*
visHexMapping, *21*, 36
visHexMulComp, *16*, *20*, *29*, 37, *42*
visHexPattern, 38
visKernels, *23*, *27*, 40
visVp, *32*, *38*, 41

Xiang, 42
