# Supplementary material for: supraHex: An R/Bioconductor package for tabular omics data analysis using a supra-hexagonal map
Source: Biochem Biophys Res Commun. 2014 Jan 3;443(1):285–9. doi: 10.1016/j.bbrc.2013.11.103 (PMC3905187; doi:10.1016/j.bbrc.2013.11.103)
Supplement: Supplementary data 2 — This document contains supplementary materials. [file mmc2.pdf]

supraHex: a supra-hexagonal map for analysing tabular omics data

Hai Fang*, Julian Gough
Department of Computer Science
University of Bristol, UK

Abstract

We introduce an R/Bioconductor package called *supraHex*[1]. It names after a supra-hexagon that is a giant hexagon on a 2-dimensional (2D) map grid seamlessly consisting of smaller hexagons. This 2D giant hexagon is intended to train, analyse and visualise a high-dimensional omics data, which usually involves tens of thousands of genomic coordinates (e.g. genes) but at most hundreds of samples. The resulting supra-hexagon ensembles the structure of the input data in a topology-preserving fashion. Thanks to the prevalence in nature and symmetric beauty, the supra-hexagon is suited for data exploratory analysis plus the ease with visualisation. In this vignette/guide, we give a tutorial-style introduction into how the functions contained in the package *supraHex* can be used to better understand the input data. This vignette assumes some basic familiarity with the `R` language and environment. It only provides a task-oriented description of the package functionality, and compliments the accompanying manual 'supraHex-manual.pdf' that gives a full description of all functions.

* hfang@cs.bris.ac.uk
[1] `http://supfam.org/SUPERFAMILY/dcGO/supraHex.html`

Contents

1 Introduction **3**
- 1.1 What is *supraHex*? . . . 3
- 1.2 Why to use *supraHex*? . . . 4
- 1.3 How to interpret *supraHex*? . . . 4

2 Installation and Citation **5**

3 Quick Overview **6**

4 Main Functionality **7**
- 4.1 Get trained using `sPipeline` . . . 8
- 4.2 Get visualised using `visHexMapping` and `visHexPattern` . . . 9
- 4.3 Get clustered using `sDmatCluster` and `visDmatCluster` . . . 10
- 4.4 Get reordered using `sCompReorder` and `visCompReorder` . . . 12

5 Comparing Neighborhood Kernels **13**

6 Applications to Real Cases **18**
- 6.1 Leukemia patient dataset from Golub et al . . . 18
- 6.2 Human embryo dataset from Fang et al . . . 18
- 6.3 Arabidopsis embryo dataset from Xiang et al . . . 20

7 Session Information **22**

List of Tables

1 `A summary of functions used for training and analysis` . . . 6
2 `A summary of functions used for visualisation` . . . 7

List of Figures

1 A supra-hexagonal map . . . 3
2 Map hit distribution . . . 9
3 Map distance visualisation . . . 10
4 Line plot of codebook patterns . . . 11
5 Bar plot of codebook patterns . . . 12
6 Clusters of the trained map . . . 13
7 Reordered components of trained map . . . 14
8 Neighborhood kernels . . . 15
9 Components of trained map with the gaussian kernel . . . 15
10 Components of trained map with the bubble kernel . . . 16
11 Components of trained map with the cutgaussian kernel . . . 16
12 Components of trained map with the ep kernel . . . 17
13 Components of trained map with the gamma kernel . . . 17
14 Reordered components of map for leukemia classification . . . 19
15 Reordered components of map during early human organogenesis 20
16 Reordered components of map during embryo development in Arabidopsis . . . 21

1 Introduction

1.1 What is *supraHex*?

The *supraHex* is a supra-hexagonal map. It consists of smaller hexagonal lattices on a regular 2-dimensional (2D) grid; these smaller hexagons collectively form a giant hexagon (see Figure 1). To be sure that a supra-hexagon is forme dexactly, inherent relationships must be met between the total number $nHex$ of hexagons in the grid, the grid radius r, and the xy-dimensions $xdim$ and $ydim$:

- $nHex = 1 + 6 * r * (r - 1)/2$;
- $xdim = ydim = 2 * r - 1$.

The codes used to produce this example are (assumedly the package has been successfully installed; see Section 2):

```
> library("supraHex")
> pdf("supraHex_vignettes-suprahex.pdf", width=6, height=6)
> sTopol <- sTopology(xdim=15, ydim=15, lattice="hexa", shape="suprahex")
> visHexMapping(sTopol,mappingType="indexes",newpage=F)
> dev.off()
```

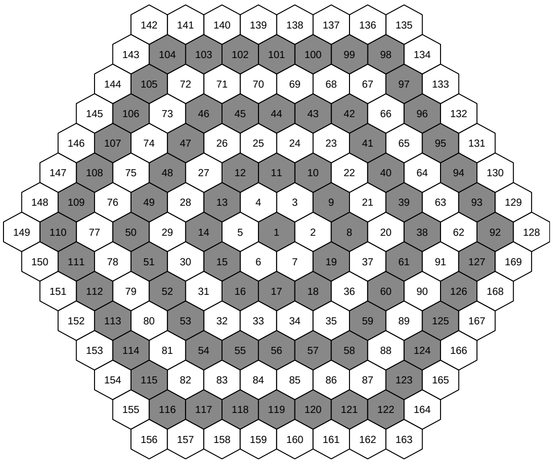

Figure 1: `A supra-hexagonal map`. It consists of $nHex = 169$ smaller hexagons. For easy reference, these hexagons are indexed according to: firstly, how many steps a hexagon is away from the grid centroid ('1' for the centroid itself); secondly, for those hexagons of the same step, an anti-clock order starting from the rightmost. This map can also be easily described by the grid radius (i.e., the maximum steps away from the centroid; $r = 8$ in this case), or by the xy-dimensions of the map grid (i.e., the maximum number of hexagons horizontally/vertically; $xdim = ydim = 15$ in this case).

1.2 Why to use *supraHex*?

Biologists are far often confronted with ever-increasing amounts of omics data that are tabulated in the form of matrix, measuring levels/activities of genomic coordinates (e.g. genes) against experimental samples. The matrix usually involves tens of thousands of genes but a much smaller number of samples (at most hundreds), known as 'small-sample-but-large-gene'. The atypical structure requires easy-to-interpret models. Unsupervised learning algorithm/model such as self-organising map is popular for its unique way in capturing input data patterns, this is, simultaneously performing vector quantisation but regularised by vector projection. The *supraHex* borrows this learning algorithm to produce a supra-hexagonal map (two-dimensional output space) from input omics data (high-dimensional input space). In this map, geographically close locations are indicative of patterns that are similar in terms of the input space. Thanks to the prevalence in nature and symmetric beauty, this supra-hexagonal map is probably suited for analysing such input data with approximately perfect symmetry. We argue that omics data tend to be symmetric due to unbiased measurements of gene levels/activities on a global scale. Even when priori knowledge of the data symmetry is unknown, we also argue that at least the supra-hexagonal map can provide the ease with visualisation.

1.3 How to interpret *supraHex*?

As a result of training, similar input data are mapped onto neighboring regions of the supra-hexagonal map. More formally, the map ensembles the structure (the shape and density) of the input data in a topology-preserving fashion. Each map node is associated with two coordinates: one in two-dimensional output space just as you have seen; the other in high-dimensional input space as you can imagine. The coordinate in input space is represented as a prototype/weight vector (with the same dimension as input data vector). Prototype vectors in all map nodes collectively form the codebook matrix. In essence, the supra-hexagonal map converts the input data into the codebook matrix. In terms of gene activity matrix as input, *supraHex* produces a map, wherein (i) genes with the same or similar activity patterns are spatially located/clustered to the same or nearby map nodes, (ii) the density of genes mapped onto this map (i.e. what we can see) is an equivalent to the data density in high-dimensional input space (i.e. what we can only imagine), and (iii) when all map nodes are color-coded according to values in a specific component for all prototype vectors (i.e. a specific column of codebook matrix), a color-coded component map can be used to illustrate sample-specific gene activities, and thus multiple components illustrate changes across all samples in subject. Owing to these unique features, the supra-hexagonal map can be used for gene clustering and sample representation.

2 Installation and Citation

supraHex is a package for the R computing environment and it is assumed that you have already installed the latest version of R. You can install the package following step-by-step guidelines in http://supfam.org/SUPERFAMILY/dcGO/supraHex.html. Briefly, you can install it from Bioconductor:

Using the release version[2] (officially released on 15-10-2013):

```
> source("http://bioconductor.org/biocLite.R")
> biocLite("supraHex")
> library(supraHex) # load the package
```

Using the development version[3] (prefer it for the benefit of latest improvements):

```
> source("http://bioconductor.org/biocLite.R")
> biocLite("BiocInstaller")
> library(BiocInstaller)
> useDevel(devel=T)
> biocLite("supraHex")
> library(supraHex) # load the package
```

To get help information for the package, please type one of two commands:

```
> library(help="supraHex") # real-time help
> help.start() # html help (follow the links to supraHex)
```

To view this vignette source and R code whereof, please type:

```
> browseVignettes("supraHex")
```

supraHex is free to use under GPL 2. You can get citation information from:

```
> citation("supraHex") # cite the package
```

[2] http://www.bioconductor.org/packages/2.13/bioc/html/supraHex.html
[3] http://bioconductor.org/packages/devel/bioc/html/supraHex.html

3 Quick Overview

The functions in the package *supraHex* are divided into two categories: one for training and analysis (see Table 1), the other for visualisation (see Table 2).

Table 1: `A summary of functions used for training and analysis`

Function	Description
`sHexGrid`	Define a supra-hexagonal grid; return a list.
`sTopology`	Define the topology of a map grid; return a `sTopol` object.
`sHexGrid`	Define a supra-hexagonal grid; return a list.
`sInitial`	Initialise a sMap object given a topology and input data; return a `sMap` object.
`sTrainology`	Define trainology (training environment); return a `sTrain` object.
`sTrainSeq`	Implement training via sequential algorithm; return a `sMap` object.
`sTrainBatch`	Implement training via batch algorithm; return a `sMap` object.
`sBMH`	Identify the best-matching hexagons for the input data; return a list.
`sPipeline`	Setup the pipeline for completing ab initio training given the input data; return a `sMap` object.
`sNeighDirect`	Calculate direct neighbors for each hexagon in a grid; return a matrix.
`sNeighAny`	Calculate any neighbors for each hexagon in a grid; return a matrix.
`sHexDist`	Calculate distances between hexagons in a 2D grid; return a matrix.
`sDistance`	Compute the pairwise distance for a given data matrix; return a matrix.
`sDmat`	Calculate distance matrix in high-dimensional input space but according to neighborhood relationships in 2D output space; return a vector.
`sDmatMinima`	Identify local minima (in 2D output space) of distance matrix (in high-dimensional input space); return a vector.
`sDmatCluster`	Partition a grid map into clusters; return a list.
`sCompReorder`	Reorder component planes; return a `sReorder` object.
`sWriteData`	Write out the best-matching hexagons and/or cluster bases in terms of data; return a data frame.
`sMapOverlay`	Overlay additional data onto the trained map; return a `sMap` object.

Table 2: `A summary of functions used for visualisation`

Function	Description
`visHexGrid`	Visualise a supra-hexagonal grid.
`visHexMapping`	Visualise various mapping items within a supra-hexagonal grid.
`visHexComp`	Visualise a component plane of a supra-hexagonal grid.
`visColormap`	Define a colormap.
`visColorbar`	Define a colorbar.
`visVp`	Create viewports for multiple supra-hexagonal grids.
`visHexMulComp`	Visualise multiple component planes of a supra-hexagonal grid.
`visCompReorder`	Visualise multiple component planes reorded within a sheet-shape rectangle grid.
`visHexPattern`	Visualise codebook matrix or input patterns within a supra-hexagonal grid.
`visDmatCluster`	Visualise clusters/bases partitioned from a supra-hexagonal grid.
`visKernels`	Visualize neighborhood kernels.

4 Main Functionality

This vignette aims to demonstrate the functionality of the package `supraHex` in utilising the supra-hexagonal map to train, analyse and visualise a high-dimensional omics data. To simplify the descriptions, it deals with the gene expression data. But it can also be applied in any other omics data, a tabular matrix usually containing thousands of genes but with at most hundreds of samples. Assumedly, we have a gene expression matrix of 1000×6, measuring the expression levels of 1000 genes across 6 samples. These samples come from two different normal distributions (S1 and S2), and each (i.e., a matrix of 1000×3) is randomly generated from the same normal distribution. All examples below are based on the latest development version[4].

```
> data <- cbind(
+ matrix(rnorm(1000*3,mean=0.5,sd=1), nrow=1000, ncol=3),
+ matrix(rnorm(1000*3,mean=-0.5,sd=1), nrow=1000, ncol=3)
+ )
> colnames(data) <- c("S1","S1","S1","S2","S2","S2")
```

The first 5 rows of this data:

```
> data[1:5,]
```

```
              S1         S1          S1          S2         S2         S2
[1,] -0.07092828 -0.1562625 -0.79243884 -0.19743561  1.1059783  0.1394123
[2,] -0.79117584  0.9928318  2.14955672 -0.13544053  0.5949748 -0.3509951
[3,]  1.88451357 -0.4786483  0.04141603  1.02633192 -1.1041313 -3.6823335
[4,] -0.69194196  0.4112544 -0.32200500 -0.09038687 -0.4576181  0.8511419
[5,]  0.82336646  0.9283436  1.51616733 -1.30519235 -0.7368782 -1.4022289
```

[4] http://bioconductor.org/packages/devel/bioc/html/supraHex.html

You can prepare your own data (a tab-delimited text file). Similarly as shown above, this file should contain the first row intended for sample names, the first column for gene names, and the top-left entry being left empty. You can import it using the R built-in function `read.table`:

```
> data <- read.table(file="you_input_data_file", header=T, row.names=1, sep="\t")
```

4.1 Get trained using `sPipeline`

The function `sPipeline` setups the pipeline for completing *ab initio* training given the input data only. It sequentially consists of:

1. `sTopology` used to define the topology of a grid (with "suprahex" shape by default) according to the input data;
2. `sInitial` used to initialise the codebook matrix given the pre-defined topology and the input data (by default using "uniform" initialisation method);
3. `sTrainology` and `sTrainSeq` used to get the grid map trained at both "rough" and "finetune" stages. If instructed, sustain the "finetune" training until the mean quantization error does get worse;
4. `sBMH` used to identify the best-matching hexagons/rectangles (BMH) for the input data, and these response data are appended to the resulting object of "sMap" class.

Below is its common usage of `sPipeline` with default setup (using gaussian kernel and printing out messages in the screen):

```
> sMap <- sPipeline(data=data)
```

Use `sWriteData` to write out the best-matching hexagons in terms of data (equivalent to gene clustering):

```
> # it will also write out a file ('Output.txt') into your disk
> output <- sWriteData(sMap=sMap, data=data, filename="Output.txt")
> output[1:5,]

  ID Hexagon_index
1  1           153
2  2           119
3  3           167
4  4            95
5  5            12
```

You will see: the first column for your input data ID (an integer; otherwise the row names of input data matrix), and the second column for the corresponding index of best-matching hexagons (i.e. gene clusters). On the way how hexagons get indexed, please refer to Figure 1.

4.2 Get visualised using `visHexMapping` and `visHexPattern`

The function `visHexMapping` is used to visualise the single-value properties that are associated with the map:

- map indexes as shown previously in Figure 1.

  ```
  > visHexMapping(sMap,mappingType="indexes",newpage=F)
  ```

- map hit distribution, which tells how many input data vectors are hitting each hexagon (see Figure 2).

  ```
  > pdf("supraHex_vignettes-hit.pdf", width=6, height=6)
  > visHexMapping(sMap,mappingType="hits",newpage=F)
  > dev.off()
  ```

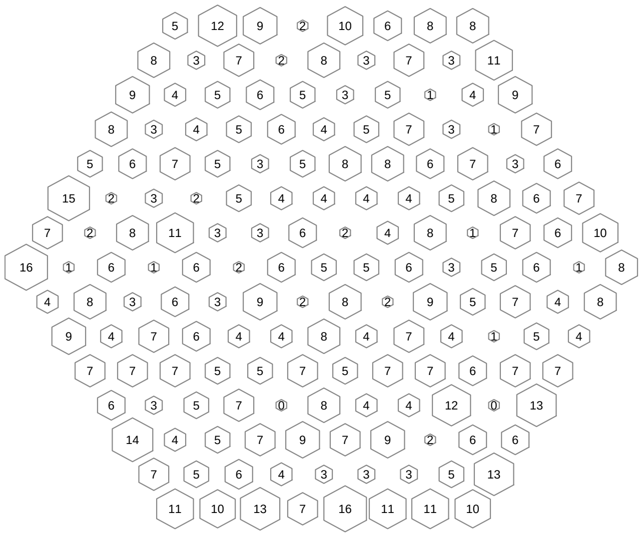

Figure 2: `Map hit distribution`. The number represents how many input data vectors are hitting each hexagon. The size of each hexagon is proportional to the number of hits.

- map distance visualisation, which tells how far each hexagon is away from its neighbors (see Figure 3).

  ```
  > pdf("supraHex_vignettes-distance.pdf", width=6, height=6)
  > visHexMapping(sMap,mappingType="dist",newpage=F)
  > dev.off()
  ```

The function `visHexPattern` is used to visualise the vector-based patterns that are associated with the map:

- using line plots (see Figure 4).

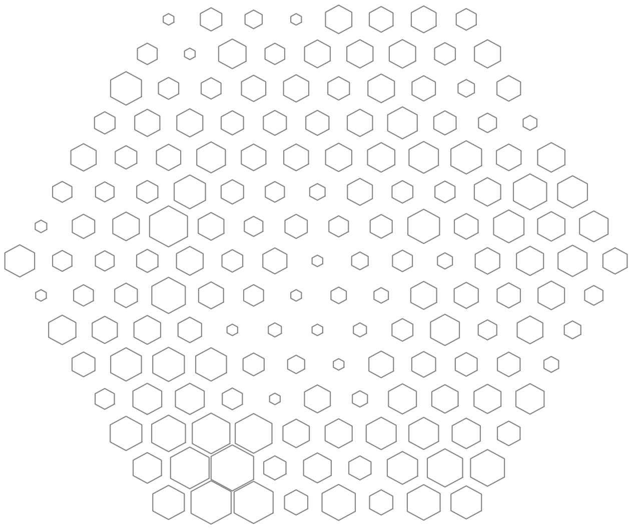

Figure 3: `Map distance visualisation`. For each hexagon, its median distances in high-dimensional input space to its neighbors (defined in 2D output space) is calculated. The size of each hexagon is proportional to this distance.

```
> pdf("supraHex_vignettes-line.pdf", width=6, height=6)
> visHexPattern(sMap, plotType="lines",
+ customized.color=rep(c("red","green"),each=3),newpage=F)
> dev.off()
```

- using bar plots (see Figure 5).

```
> pdf("supraHex_vignettes-bar.pdf", width=6, height=6)
> visHexPattern(sMap, plotType="bars",
+ customized.color=rep(c("red","green"),each=3),newpage=F)
> dev.off()
```

Both functions also support the visualisation of user-customised data. On this advanced usage, please refer to specifications of functions by:

```
> ?visHexMapping
> ?visHexPattern
```

4.3 Get clustered using `sDmatCluster` and `visDmatCluster`

Partition the trained map into clusters using region-growing algorithm to ensure each cluster is continuous (see Figure 6).

```
> sBase <- sDmatCluster(sMap=sMap, which_neigh=1,
+ distMeasure="median", clusterLinkage="average")
```

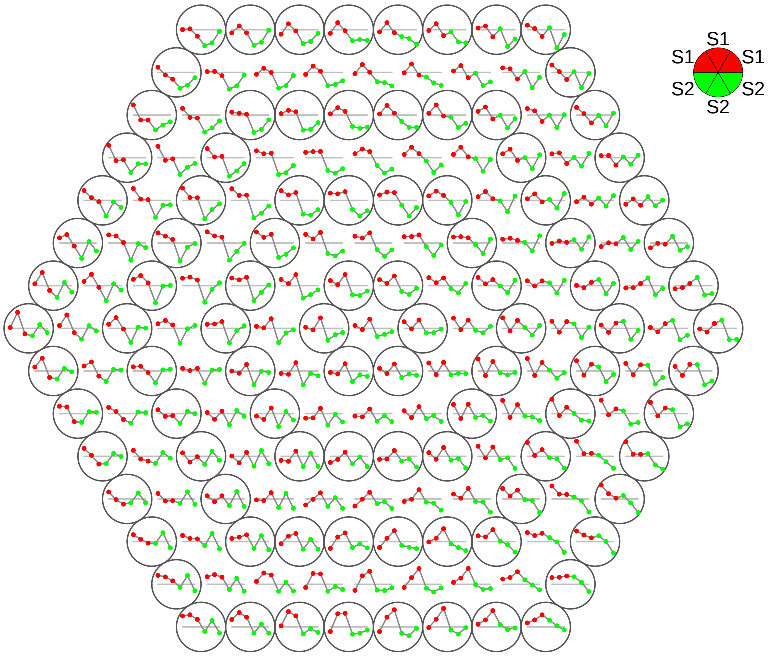

Figure 4: `Line plot of codebook patterns`. If multple colors are given, the points are also plotted. When the pattern involves both positive and negative values, zero horizental line is also shown.

```
> pdf("supraHex_vignettes-cluster.pdf", width=6, height=6)
> visDmatCluster(sMap, sBase, newpage=F)
> dev.off()
```

It is equivalent to gene meta-clustering. Write out results into a tab-delimited text file using `sWriteData`:

```
> # it will also write out a file ('Output_base.txt') into your disk
> output <- sWriteData(sMap, data, sBase, filename="Output_base.txt")
> output[1:5,]

  ID Hexagon_index Cluster_base
1  1           153           13
2  2           119           15
3  3           167           17
4  4            95            5
5  5            12            3
```

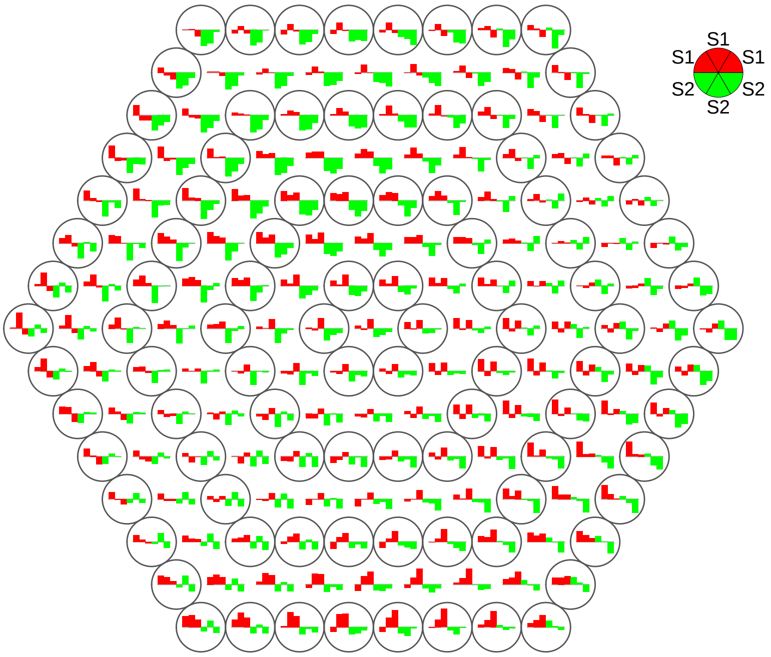

Figure 5: `Bar plot of codebook patterns`. When the pattern involves both positive and negative values, the zero horizontal line is in the middle of the hexagon; otherwise at the top of the hexagon for all negative values, and at the bottom for all positive values.

You will see: the first column for your input data ID (an integer; otherwise the row names of input data matrix), the second column for the corresponding index of best-matching hexagons (i.e. gene clusters), and the third column for the cluster bases (i.e. gene meta-clusters).

4.4 Get reordered using `sCompReorder` and `visCompReorder`

Reordering components for trained map can be realised by using a new map grid (with sheet shape consisting of a rectangular lattice) to train component plane vectors (either column-wise vectors of codebook/data matrix or the covariance matrix thereof). As a result, similar component planes are placed closer to each other. The functions `sCompReorder` and `visCompReorder` are respectively to implement this reordering algorithm and to visualise the reordered compments (see Figure 7)[5].

[5]In order to display colors properly, it is important to reset the argument 'zlim' by respecting the range of input data matrix (more precisely, codebook matrix). By default, it is set to

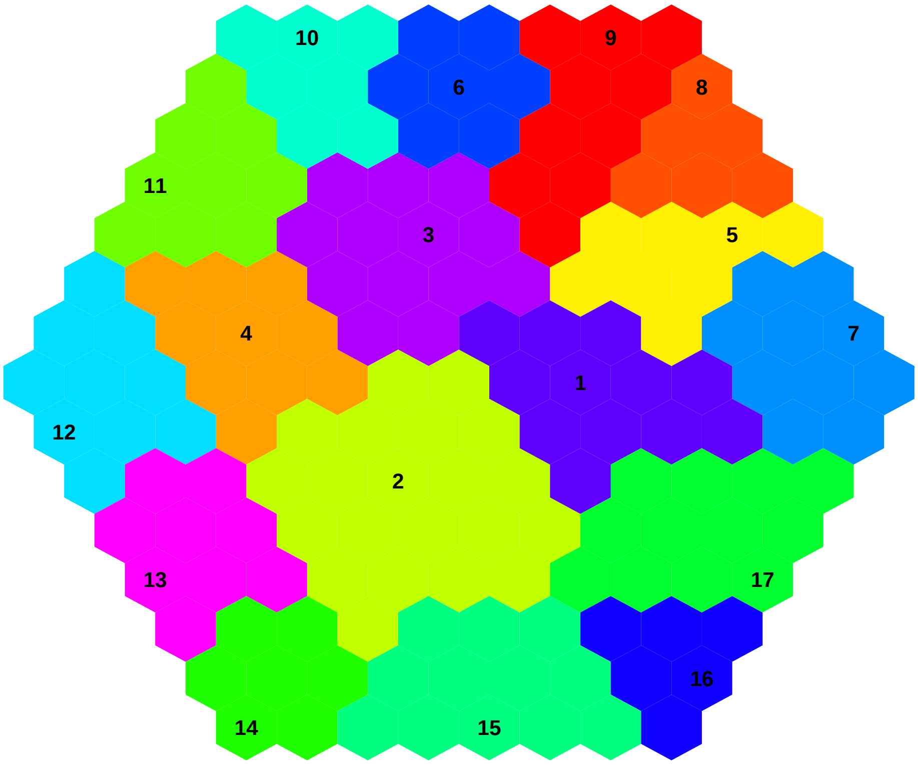

Figure 6: `Clusters of the trained map`. Each cluster is filled with the same continuous color. The cluster index is marked in the seed node.

```
> sReorder <- sCompReorder(sMap=sMap)
> visCompReorder(sMap=sMap, sReorder=sReorder)
```

It is equivalent to sample projection. Reordered components are rich in the information: both of genes and samples can be visualised but in a single display.

5 Comparing Neighborhood Kernels

Among various parameters associated with the training by `sPipeline`, the neighborhood kernel is the most important one because it dictates the final topology of the trained map. For visualising neighborhood kernels, the function `visKernels` helps to understand their forms (see Figure 8). Each kernel is a

c(0,1). The simplest way is to first choose the range from the global minimum of your input data matrix to the global maximum, and then to fine-tune this choose for the best display.

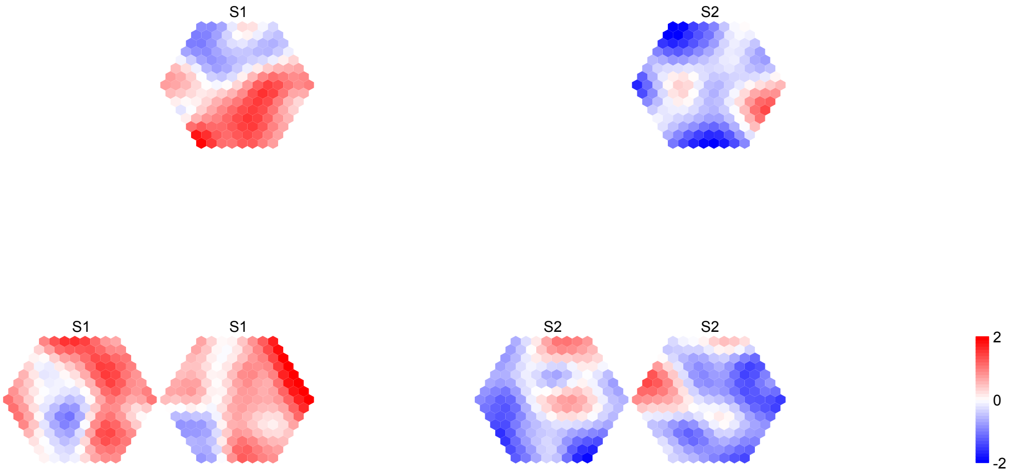

Figure 7: `Reordered components of trained map`. Each component illustrates the sample-specific map and is placed within a two-dimensional rectangular lattice (framed in black). Within each component, genes with the same or similar expression patterns are mapped to the same or nearby map nodes. When zooming out to look at between-components/samples relationships, samples with the similar expression profiles are placed closer to each other.

non-increasing functions of: i) the distance between the hexagon/rectangle and the winner, and ii) the radius.

```
> pdf("supraHex_vignettes-kernels.pdf", width=12, height=6)
> visKernels(newpage=F)
> dev.off()
```

From the mathematical definitions and curve forms above, it is clear that the "gamma" and "gaussian" kernels exert more global influence, the "ep" kernel puts more emphasis on local topological relationships, and the other two "cutgaussian" and "bubble" keep the relative balance. It becomes much clearer when using the function `visHexMulComp` to visualise trained maps using the same data input and the same trainology but choosing different kernels (see Figure 9, Figure 10, Figure 11, Figure 12 and Figure 13)[6].

- with "gaussian" kernel (see Figure 9)

```
> sMap_ga <- sPipeline(data=data, neighKernel="gaussian", init="uniform")
> visHexMulComp(sMap_ga)
```

[6]In order to display colors properly, it is important to reset the argument 'zlim' by respecting the range of input data matrix (more precisely, codebook matrix). The simplest way is to first choose the range from the global minimum of your input data matrix to the global maximum, and then to fine-tune this choose for the best display.

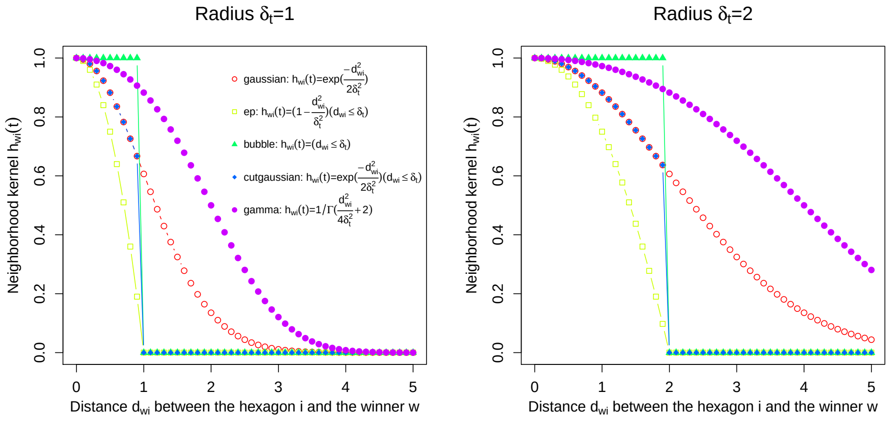

Figure 8: `Neighborhood kernels`. There are five kernels that are currently supported in the package *supraHex*. These kernels are displayed within a plot for each fixed radius; two different radii (i.e. 1 and 2) are illustrated only.

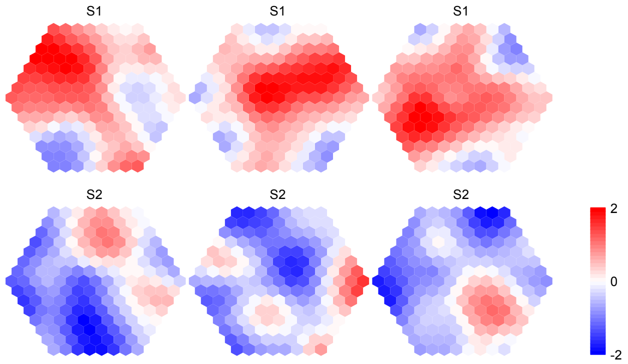

Figure 9: `Components of trained map with the gaussian kernel`.

- with "bubble" kernel (see Figure 10)

```
> sMap_bu <- sPipeline(data=data, neighKernel="bubble", init="uniform")
> visHexMulComp(sMap_bu)
```

- with "cutgaussian" kernel (see Figure 11)

```
> sMap_cu <- sPipeline(data=data, neighKernel="cutgaussian", init="uniform")
> visHexMulComp(sMap_cu)
```

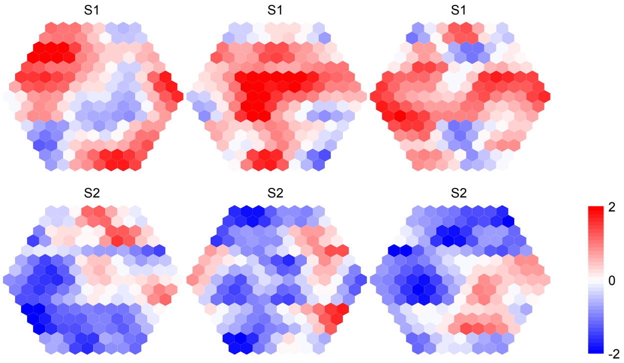

Figure 10: `Components of trained map with the bubble kernel`.

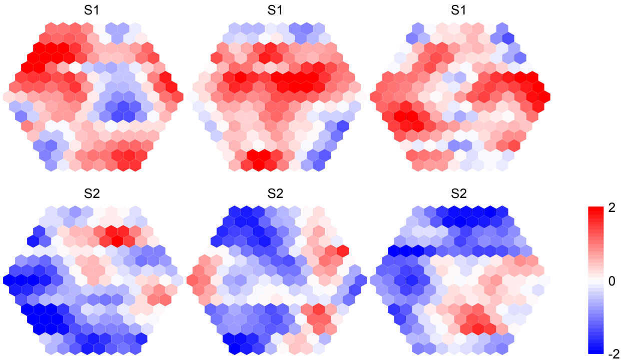

Figure 11: `Components of trained map with the cutgaussian kernel`.

- with "ep" kernel (see Figure 12)

```
> sMap_ep <- sPipeline(data=data, neighKernel="ep", init="uniform")
> visHexMulComp(sMap_ep)
```

- with "gamma" kernel (see Figure 13)

```
> sMap_gm <- sPipeline(data=data, neighKernel="gamma", init="uniform")
> visHexMulComp(sMap_gm)
```

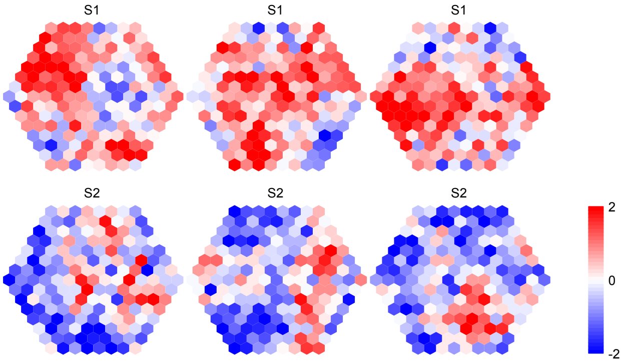

Figure 12: `Components of trained map with the ep kernel`.

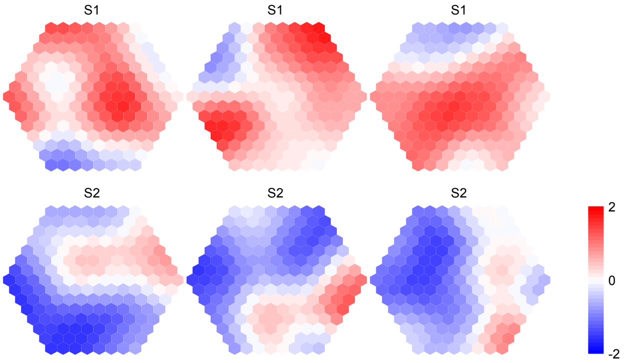

Figure 13: `Components of trained map with the gamma kernel`.

6 Applications to Real Cases

The most common real cases are applications in studies involving gene expression profilings of: i) clinical patients; ii) time-course processes. In this section, we aim to showcase the applications by providing several datasets published previously and a collection of functions (together with optimised arguments) to analyse them. The end users are encouraged to adapt them to fit your dataset: run them first, then get down to details. We do not repeat the explanations for all used commands and output files and figures. On the meanings and interpretations, please refer to Section 4). On purpose of easy copying, all commands are provided without the '>' prefix:

6.1 Leukemia patient dataset from Golub et al

This dataset (the learning set[7]) contains a 3051×38 matrix of expression levels, involving 3051 genes and two types of leukemia: 11 acute myeloid leukemia (AML) and 27 acute lymphoblastic leukemia (ALL). These 27 ALL are further subtyped into 19 B-cell ALL (ALL_B) and 8 T-cell ALL (ALL_T) (see Figure 14).

```
# import data
data(Golub)
data <- Golub
# get trained
sMap <- sPipeline(data)
visHexMulComp(sMap,title.rotate=10,
colormap="darkgreen-lightgreen-lightpink-darkred")
sWriteData(sMap, data, filename="Output_Golub.txt")
# get visualised
visHexMapping(sMap, mappingType="indexes")
visHexMapping(sMap, mappingType="hits")
visHexMapping(sMap, mappingType="dist")
# get reordered
sReorder <- sCompReorder(data,metric="pearson")
visCompReorder(sMap,sReorder,title.rotate=15,
colormap="darkgreen-lightgreen-lightpink-darkred")
```

6.2 Human embryo dataset from Fang et al

This dataset[8] involves six successive developmental stages with three replicates for each stage (see Figure 15), including:

- Fang: a 5441×18 matrix of expression levels;
- Fang.geneinfo: a 5441×3 matrix of gene information;
- Fang.sampleinfo: a 5441×3 matrix of sample information.

```
# import data
data(Fang)
```

[7] http://www.ncbi.nlm.nih.gov/pubmed/10521349
[8] http://www.ncbi.nlm.nih.gov/pubmed/20643359

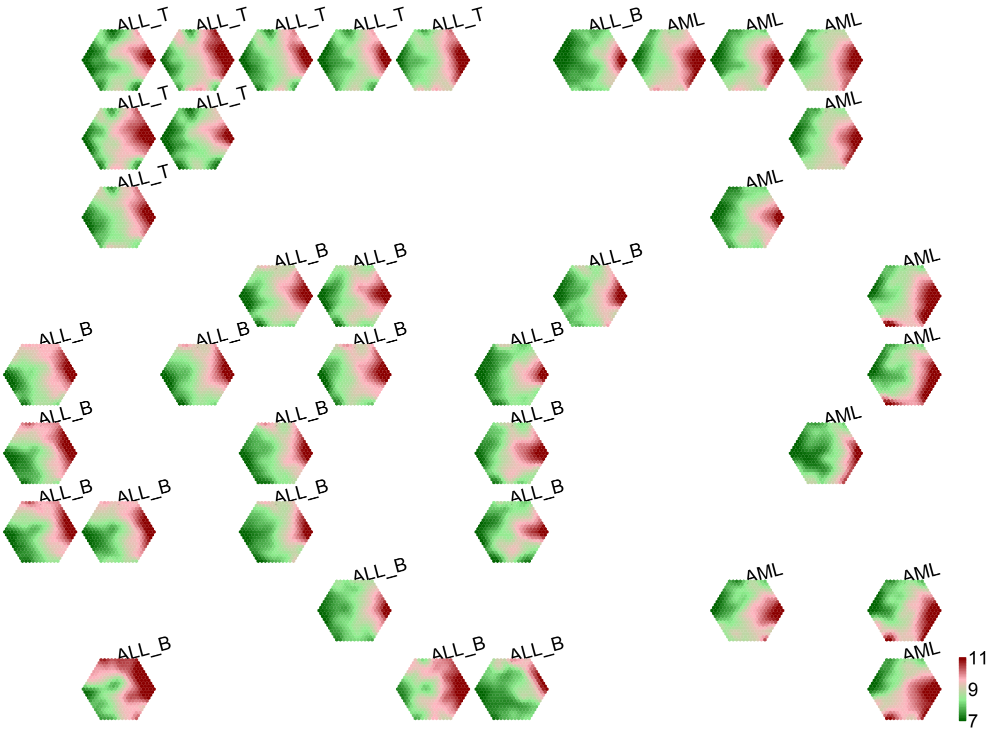

Figure 14: `Reordered components of map for leukemia classification.` Each component illustrates a sample-specific transcriptome map. Geometric locations of components display the relationships between 38 leukemia samples. AML: acute myeloid leukemia; ALL: acute lymphoblastic leukemia; ALL_B: B-cell ALL; ALL_T: T-cell ALL.

```
# transform data by row/gene centering
data <- Fang - matrix(rep(apply(Fang,1,mean),ncol(Fang)),ncol=ncol(Fang))
# get trained
sMap <- sPipeline(data)
visHexMulComp(sMap,title.rotate=10)
sWriteData(sMap, data, filename="Output_Fang.txt")
# get visualised
visHexMapping(sMap, mappingType="indexes")
visHexMapping(sMap, mappingType="hits")
visHexMapping(sMap, mappingType="dist")
visHexPattern(sMap, plotType="lines")
visHexPattern(sMap, plotType="bars")
# get clustered
sBase <- sDmatCluster(sMap)
visDmatCluster(sMap, sBase)
sWriteData(sMap, data, sBase, filename="Output_base_Fang.txt")
```

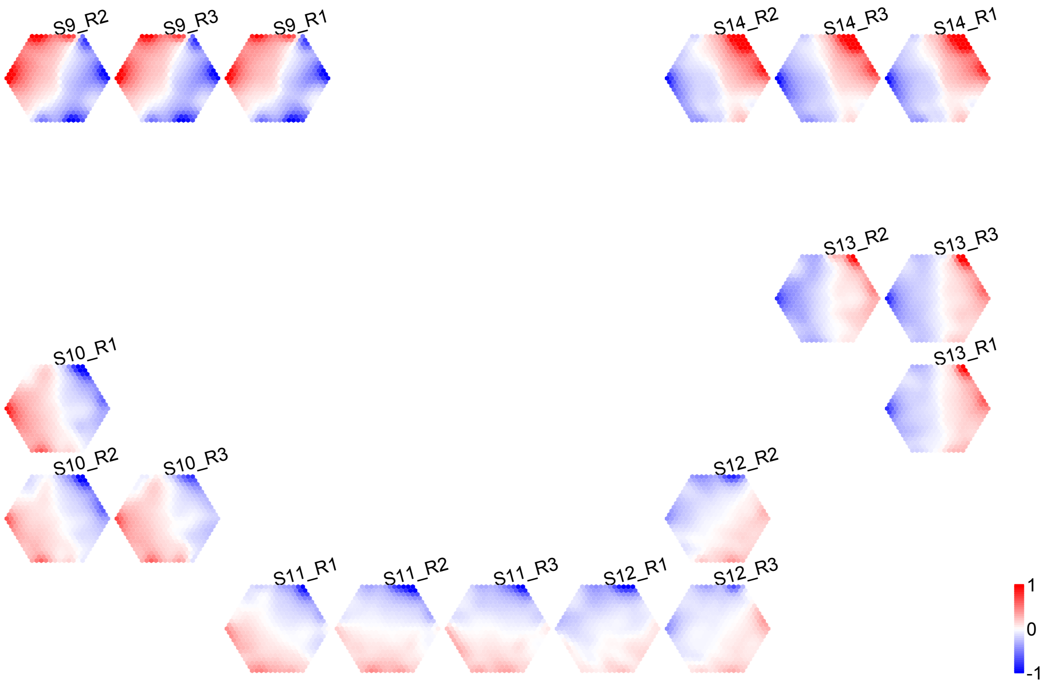

Figure 15: `Reordered components of map during early human organogenesis`. Each component illustrates a sample-specific transcriptome map. Geometric locations of components display the relationships between the six developmental stages (S9-S14), each with three replicates (R1-R3).

```
# get reordered
sReorder <- sCompReorder(data, metric="euclidean")
visCompReorder(sMap, sReorder,title.rotate=15)
```

6.3 Arabidopsis embryo dataset from Xiang et al

This dataset[9] contains gene expression levels (3625 genes and 7 embryo stages) (see Figure 16).

```
# import data
data(Xiang)
data <- Xiang
# get trained
sMap <- sPipeline(data)
visHexMulComp(sMap,title.rotate=10,colormap="darkblue-white-darkorange")
sWriteData(sMap, data, filename="Output_Xiang.txt")
```

[9] http://www.ncbi.nlm.nih.gov/pubmed/21402797

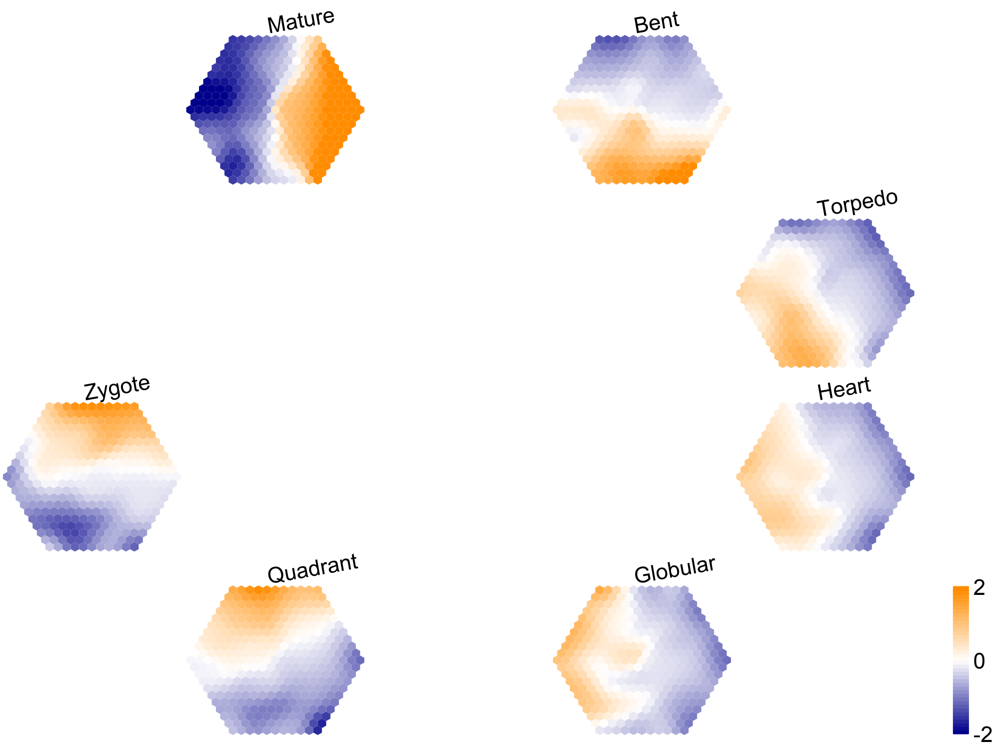

Figure 16: `Reordered components of map during embryo development in Arabidopsis`. Geometric locations of sample-specific transcriptome map characterise the relationships between the seven developmental stages: zygote, quadrant, globular, heart, torpedo, bent and mature.

```
# get visualised
visHexMapping(sMap, mappingType="indexes")
visHexMapping(sMap, mappingType="hits")
visHexMapping(sMap, mappingType="dist")
visHexPattern(sMap, plotType="lines")
visHexPattern(sMap, plotType="bars")
# get clustered
sBase <- sDmatCluster(sMap)
visDmatCluster(sMap, sBase)
sWriteData(sMap, data, sBase, filename="Output_base_Xiang.txt")
# get reordered
sReorder <- sCompReorder(sMap, metric="pearson")
visCompReorder(sMap,sReorder,title.rotate=10,colormap="darkblue-white-darkorange")
```

7 Session Information

All of the output in this vignette was produced under the following conditions:

```
> sessionInfo()

R version 3.0.2 (2013-09-25)
Platform: x86_64-apple-darwin10.8.0 (64-bit)

locale:
[1] C/en_GB.UTF-8/en_GB.UTF-8/C/en_GB.UTF-8/en_GB.UTF-8

attached base packages:
[1] grid      stats     graphics  grDevices utils     datasets  methods
[8] base

other attached packages:
[1] supraHex_1.1.3  hexbin_1.26.2   lattice_0.20-24

loaded via a namespace (and not attached):
[1] MASS_7.3-29 tools_3.0.2
```
